# Rapamycin Reverses Status Epilepticus-Induced Memory Deficits and Dendritic Damage

**DOI:** 10.1371/journal.pone.0057808

**Published:** 2013-03-11

**Authors:** Amy L. Brewster, Joaquin N. Lugo, Vinit V. Patil, Wai L. Lee, Yan Qian, Fabiola Vanegas, Anne E. Anderson

**Affiliations:** 1 Cain Foundation Laboratories, Jan and Dan Duncan Neurological Research Institute at Texas Children’s Hospital and Department of Pediatrics, Baylor College of Medicine, Houston, Texas, United States of America; 2 Department of Neurology, Baylor College of Medicine, Houston, Texas, United States of America; 3 Department of Neuroscience, Baylor College of Medicine, Houston, Texas, United States of America; University of Texas Health Science Center, United States of America

## Abstract

Cognitive impairments are prominent sequelae of prolonged continuous seizures (status epilepticus; SE) in humans and animal models. While often associated with dendritic injury, the underlying mechanisms remain elusive. The mammalian target of rapamycin complex 1 (mTORC1) pathway is hyperactivated following SE. This pathway modulates learning and memory and is associated with regulation of neuronal, dendritic, and glial properties. Thus, in the present study we tested the hypothesis that SE-induced mTORC1 hyperactivation is a candidate mechanism underlying cognitive deficits and dendritic pathology seen following SE. We examined the effects of rapamycin, an mTORC1 inhibitor, on the early hippocampal-dependent spatial learning and memory deficits associated with an episode of pilocarpine-induced SE. Rapamycin-treated SE rats performed significantly better than the vehicle-treated rats in two spatial memory tasks, the Morris water maze and the novel object recognition test. At the molecular level, we found that the SE-induced increase in mTORC1 signaling was localized in neurons and microglia. Rapamycin decreased the SE-induced mTOR activation and attenuated microgliosis which was mostly localized within the CA1 area. These findings paralleled a reversal of the SE-induced decreases in dendritic Map2 and ion channels levels as well as improved dendritic branching and spine density in area CA1 following rapamycin treatment. Taken together, these findings suggest that mTORC1 hyperactivity contributes to early hippocampal-dependent spatial learning and memory deficits and dendritic dysregulation associated with SE.

## Introduction

Prolonged continuous seizure activity (status epilepticus, SE) is associated with significant long-term cognitive impairments that include deficits in memory, attention, executive function, verbal fluency, and social perception [1]. Although cognitive impairments are observed in humans and animal models following SE [1], [2], [3], [4], the underlying molecular mechanisms remain elusive. Research in cognitive neuroscience has shown that excessive activation of the mammalian target of rapamycin complex 1 (mTORC1) pathway in transgenic rodents is associated with behavioral deficits, which are reversed following treatment with the mTOR inhibitor rapamycin [5], [6], [7]. In rodent models, an episode of SE triggers an immediate and long-lasting increase in the phosphorylation of the S6 ribosomal protein in the hippocampus [8], [9], which is a downstream marker of mTORC1 activation [10], [11]. Therefore, aberrant mTORC1 activation may be a candidate mechanism underlying the hippocampal-dependent memory deficits associated with SE.

Mammalian target of rapamycin (mTOR) is a serine/threonine kinase that is downstream in the phosphatidylinositol 3-kinase (PI3K) signaling cascade, and has ubiquitous expression in a broad range of cells. Association of mTOR with raptor (regulatory associated protein of mTOR) or rictor (rapamycin-insensitive companion of mTOR) activates the mTORC1 or mTORC2 complex, respectively. Activation of mTORC1 phosphorylates its downstream targets, including the ribosomal S6 Kinases 1/2 (S6K1/2) (upstream regulator of the S6 ribosomal protein) and the eukaryotic initiation factor 4E-binding protein (4EBP). Signaling through these proteins contributes to the translational machinery, and therefore regulates protein synthesis [10], [11]. In the brain, mTOR signaling plays a role in diverse neuronal and glial functions. In hippocampal neurons, mTORC1 modulates synaptic plasticity [10], [11], expression of voltage-gated potassium channels [12], [13], Map2 [14], and dendritic architecture [15], [16], while in microglia it modulates pro-inflammatory activation and proliferation (microgliosis) [17], [18]. Activation of mTORC2 leads to phosphorylation of AKT (S473) and other targets which regulate cell survival, proliferation, and organization of the actin cytoskeleton [19].

Given the physiological functions of mTOR signaling it is possible that dysregulation of this pathway plays a role in SE-induced cognitive deficits. Indeed, recent studies in rodent models of seizures and epilepsy have shown that suppression of SE-induced mTOR hyperactivity with rapamycin prevents or reverses some of the cellular and molecular alterations implicated in epileptogenesis such as mossy fiber sprouting, cell death, neurogenesis, and seizure activity [8], [9]. However, the effects of rapamycin on the behavioral deficits and dendritic pathology associated with SE have not been evaluated.

Spatial learning and memory deficits and hippocampal mTOR hyperactivation have been reported following SE in rodents [2], [3], [8], [9]. Furthermore, previous studies suggest a correlation between spatial memory impairments and dendritic alterations such as aberrant dendritic structure [20], [21], [22], [23] and Kv channel expression [24], [25], [26]. Thus, we tested the hypothesis that mTORC1 dysregulation plays a role in early hippocampal-dependent memory deficits and dendritic pathology following pilocarpine-induced SE. In parallel, we characterized cell types involved in the aberrant mTORC1 signaling that results following SE.

## Materials and Methods

### Ethics Statement

All procedures concerning animals were approved by the Baylor College of Medicine Institutional Animal Care and Use Committee and conformed to NIH guidelines.

### Animals

Male Sprague Dawley rats were housed at the Baylor College of Medicine Center for Comparative Medicine at an ambient temperature of 22°C, with a 14-hour (hr) light and 10-hr dark (20∶00 to 06∶00 hr) diurnal cycle and were given unlimited access to food and water.

### Pilocarpine-induced Status Epilepticus

Adult rats were injected with scopolamine methylbromide (1 mg/kg) intraperitoneally (i.p.) to minimize the effects of enhanced cholinergic activation peripherally. Thirty minutes (min) after scopolamine, pilocarpine (280 mg/kg; Sigma Chemical Co., St Louis, MO, USA) or saline (Sham) was injected (i.p.). Behavioral or video-electroencephalogram (vEEG) observation was used to monitor seizure activity and scoring was performed according to the Racine scale [27]. The onset of SE was determined by onset of class 5 limbic motor seizures (rearing and falling). SE was allowed to continue for up to 1 hr, at which point rats were given subcutaneous (s.c.) injections of sodium pentobarbital (PB; 30 mg/kg; Sigma Chemical Co.) to stop seizure activity. Two hours after PB injections, rats were given s.c. injections of 5% dextrose and 0.9% saline (2 ml/rat) (B. Braun Medical Inc. Irvine, CA, USA) for hydration and were monitored daily for adequate food and water intake. Approximately 90% of rats subjected to pilocarpine-induced SE survived and were used for the subsequent behavioral tests and monitoring for seizures and epileptiform activity.

### Rapamycin Treatment

**Figure 1:**
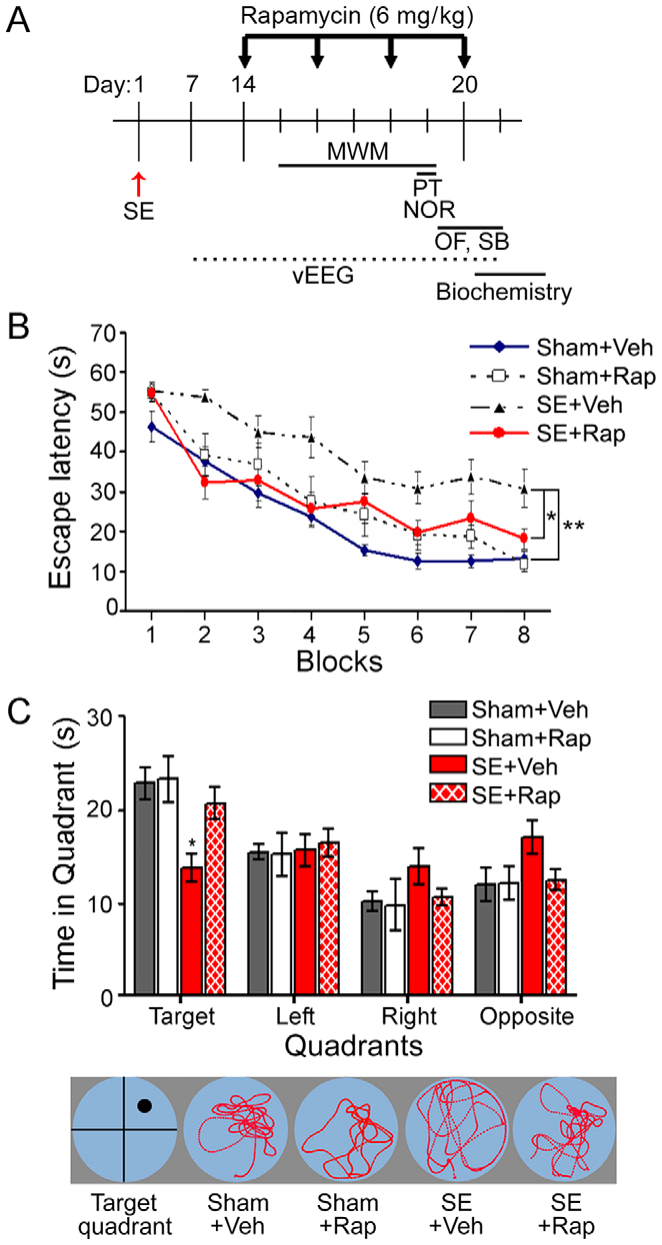
Rapamycin improved spatial learning and memory in rats subjected to SE. (**A**) The timeline of SE induction (day 1), rapamycin (Rap) treatment (6 mg/kg; days 14, 16, 18 and 20), Morris water maze (MWM) training (days 15–19), probe test (PT) (day 19), novel object recognition (NOR) (day 19) (Fig. 2), social behavior (SB) and open field (OF) (days 20–21), video-electroencephalogram (vEEG) recordings (between 7–21 days after SE), and hippocampi dissection for biochemical analyses is shown. (**B–C**) MWM results for the four experimental groups. (**B**) The rats were trained over 8 blocks (2/day, 4 days) to find a hidden platform (target quadrant) in opaque water using visual cues (acquisition phase). The time to find the hidden platform (escape latency) was significantly longer in the SE+Veh compared to the Sham+Veh group. There was no difference in the SE+Rap, Sham+Veh, and Sham+Rap groups. (**C**, top panel) The hidden platform was removed, and the rats were tested for memory retention of the target quadrant (probe trial). The SE+Veh group spent significantly less time in the target quadrant compared to the Sham+Veh group. The SE+Rap group performed similar to the Sham+Veh and Sham+Rap groups. (**C**, bottom panel) Representative tracking of the swim patterns during the probe trial. *P<0.05; **P<0.01, ANOVA with Tukey’s *post hoc* test; n = 8−12. Error bars = SEM.

To inhibit signaling of the mTORC1 pathway, rats were given rapamycin (i.p.) (LC laboratories, Woburn, MA, USA). Because of solubility issues, a rapamycin (Rap) solution at a concentration of 3 mg/ml was prepared using a diluent consisting of 4% ethanol/5% polyethylene glycol-400 (PEG-400)/5% Tween 80, as previously described [28]. This solution was made fresh 30 min prior to injecting rats. Rats were randomly selected to receive rapamycin (6 mg/kg) (Sham+Rap or SE+Rap) or vehicle (Veh; 4% ethanol/5% PEG-400/5% Tween 80) (Sham+Veh or SE+Veh). Treatments started 2 weeks after pilocarpine-induced SE and continued every other day for a total of 4 treatments per rat. Injections were administered after 4 pm. See Fig. 1A for overview of design.

### Morris Water Maze

Two weeks after pilocarpine-induced SE, the Morris water maze (MWM) was used to examine hippocampal-dependent spatial learning and memory as previously described [29]. A 1.6 meter (m) diameter circular pool was filled with water made opaque by the addition of a blue non-toxic paint. A square platform (14.5×14.5 cm) was placed approximately 2 cm below the surface of the water. Then, rats were tested for their ability to locate the hidden platform. The movement of the rats was monitored by a video camera connected to a digital tracking device (Noldus Information Technology, Leesburg, VA, USA). The MWM was conducted over a total of 4 days with 2 blocks of trials per day and 4 trials per block. The four trials per block were averaged for each block for the data analysis. After the 8^th^ block each rat was given a probe trial. In the probe trial, the platform was removed and each rat was allowed 60 seconds (s) to search the pool for the platform using visual cues in the room that were unchanged from the training phase. The amount of time that each rat spent in each quadrant was recorded (quadrant time). The following day a visible platform test was conducted to evaluate whether the rats had difficulty in locating a visible platform, as an inability or reduced ability to find the visible platform could be due to a visual or motor deficit. The data were analyzed using a mixed-design One Factor Analysis of Variance (ANOVA) (>2 treatment groups). Subsequent one-way ANOVAs were conducted if an interaction between group and block or quadrant in the probe trial was found. The escape latency for the visible platform testing was recorded for four trials averaged within each subject and then analyzed across groups using a one-way ANOVA (n = 8−12, Sham+Veh: 11, Sham+Rap: 8, SE+Veh: 12, SE+Rap: 12).

### Novel Object Recognition Test

The novel object recognition (NOR) test was used to assess recognition memory in SE and sham rats treated with at least 3 doses of rapamycin or vehicle before the test (see diagram in Fig. 1A). The NOR test was performed as previously described [30] with a lapse of 2 hrs between training and testing. Rats were habituated in the test chamber (40×40×30 cm) for 20 min. The following day, rats from all groups were placed back in the same chamber which contained two similar objects. Rats were allowed to freely explore the objects for a total of 5 minutes and then were returned to their home cages. Two hrs later, one previously explored object was replaced with a new (novel) object. Rats were placed back in the testing chamber with the novel object and one previously explored object (familiar object) for 5 minutes. Rats were evaluated for their ability to remember the familiar object. Object exploration was defined as the time rats were in direct contact with the object (e.g. sniffing of the objects, <1 cm from objects). Between each trial, the chamber and objects were thoroughly cleaned with 30% isopropyl alcohol then dried with paper towels. The data were analyzed using ANOVA with Tukey’s *post hoc* test (n = 7−11; Sham+Veh: 7, Sham+Rap: 8, SE+Veh: 10, SE+Rap: 11).

### Video-Electroencephalogram (vEEG) Recordings

Behavioral and vEEG recordings were used to monitor seizure and epileptiform activity. Rats were implanted with cortical and hippocampal electrodes as previously described [31]. Briefly, rats were anaesthetized using a Ketamine/Xylazine/Acepromazine mixture and then positioned in a stereotaxic frame. Cortical electrodes were placed bilaterally over the somatosensory cortex, hippocampi and in the cervical paraspinous area (ground electrode). All the coordinates were determined with reference to bregma. The coordinates were as follows: Hippocampi: medio-lateral, left = +2.75 mm, right = −2.75 mm, antero-posterior = −3.8 mm, and dorso-ventral = −2.75 mm. Cortex: medio-lateral, left = +2.75 mm, right = −2.75 mm, antero-posterior = −1 mm, and dorso-ventral = −1 mm. The ground electrode was placed in the paraspinous region and the reference was placed over the surface of the frontal lobe at the depth of 1/2 mm. Rats were allowed to recover for a period of 7–9 days, and then we recorded baseline vEEG activity. One week after, we administered pilocarpine or saline. Four channels of EEG activity with synchronized video were recorded using a digital Stellate system (Natus Medical Inc., San Carlos, CA, USA) or Nicolet system (NicView 5.71, CareFusion, San Diego, CA, USA). All rats were recorded one week before the induction of SE to determine baseline EEG activity. One week following the pilocarpine-induction of SE, all rats were monitored daily for behavioral seizures. Between 1–3 weeks after SE, vEEG was used to monitor electrographic activity in sessions of 4–5 hrs/day for a total of 384 hrs of vEEG recordings (n = 15−16). Two weeks after SE, rats were selected randomly to be treated with rapamycin or vehicle (n = 7−8/group). Interictal spike activity, defined as short-duration (<200 ms) fast epileptiform activity of high amplitude transients (two times baseline), was evaluated and counted in 6 epochs of 10 s during wakefulness [9], [32]. The entire EEG traces were examined for seizure activity defined as repetitive spike and slow wave discharges of 10 s or longer. Investigators blind to the conditions scored the EEG traces (AEA, ALB, VVP and WLL). Student’s t test or One-way ANOVA with Tukey’s *post hoc* test were used to determine significance between SE rats treated with rapamycin or vehicle in cortex (n = 7−8) and also in hippocampus (n = 3). Assessments included pre-treatment and following 1, 2 or 3 treatments with vehicle or rapamycin. In addition, the cohort of rats tested in the MWM, NOR, SB, and OF tasks were monitored for behavioral seizures during weeks 2–3 for ∼8 hrs/day (SE+Veh, n = 29; SE+Rap, n = 31).

### Western Blotting

Rats were decapitated 2 (n = 3−5) and 3 weeks (n = 5−8) after the induction of SE. Hippocampi were rapidly dissected, rinsed in 1X PBS, and placed on dry ice. All samples were stored at −80°C until used. Hippocampi were homogenized in ice-cold homogenization buffer (100 mM Tris-HCl, pH 7.4, 0.32 M sucrose, 1 mM EDTA, 5 mM Hepes) containing protease inhibitor cocktail (Roche, Alameda, CA, USA) and processed for western blotting as previously described [31]. The protein concentration was determined using the Bradford Protein Assay (Bio Rad, Hercules, CA, USA). The samples were normalized and diluted in Laemmli loading buffer (4X: 0.25 M Tris, pH 6.8, 6% SDS, 40% Sucrose, 0.04% Bromophenol Blue, 200 mM Dithiothreitol). Following SDS-PAGE, proteins were transferred to Hybond-P polyvinyl difluoride membranes (GE Healthcare, Piscataway, NJ, USA). Membranes were incubated in blocking solution [5% non-fat milk diluted in 1X Tris Buffered Saline (50 mM Tris-HCl, pH 7.4, 150 mM NaCl) with 0.1% Tween (1X TBS-T) and 1 mM Na_3_VO_4_] for 1 hour at room temperature (RT). Membranes were then incubated overnight at 4°C with the primary antibodies diluted in blocking solution. The primary antibodies used were as follows: Ribosomal S6 protein, P-S6 (S240/244), P-S6 (S235/236), 4EBP1, P-4EBP1 (T37/46), AKT, P-AKT (S473) (1∶2K; Cell Signaling Technology, Boston, MA, USA); Kv4.2, Kv1.1, Kv1.2, Kv1.4, HCN1, HCN2 (1∶1K; NeuroMab, Davis, CA, USA); Map2 (1∶10K; Millipore, Temecula, CA, USA); Synaptophysin, Gad67 (1∶10K; Chemicon, Billerica, MA, USA); SK2 (1∶1K; Alomone labs, Israel), and actin (1∶5K; Sigma Chemical Co.). Following incubation in primary antibodies membranes were washed in 1X TBS-T (3×5 min). Membranes were then incubated with horseradish peroxidase labeled secondary antibodies: goat anti-rabbit IgG or anti-mouse IgG (1∶5K; Cell Signaling Technology, Boston, MA, USA). After washes in 1X TBS-T membranes were incubated with Pierce enhanced chemiluminescence (Thermo Scientific, Rockford, IL, USA) and immunoreactive bands were captured on autoradiography film (Blue X-Ray Film, Phoenix Research, Candler, NC, USA). The films were developed and densitized using a Hewlett Packard Scanjet (Palo Alto, CA, USA). In some cases, membranes were stripped from primary antibodies by incubating in stripping buffer (25 mM glycine, pH 2.0, 10% SDS) for 30 min at 50°C. Membranes were then washed in 1X TBS-T (3×10 min), blocked, and re-incubated with different primary antibodies as described above.

### Western Blot Analyses

Optical density of immunoreactive bands was measured using the Image J software (NIH; Bethesda, MD, USA) as described previously [31]. Optical densities obtained for all bands from phospho-proteins were normalized to the levels of the total protein from the same sample, and expressed as ratio of phospho to-total protein. Optical densities obtained for all bands of interest were normalized for loading to the actin levels within the same lane. Each experimental point represents a single rat (n = 1). All groups were normalized to the average of the control group (Sham+Veh). ANOVA with the Tukey’s *post hoc* test was used to determine significance between groups. We are aware of the presence of non-specific signal caused by the commercially available SK2 antibody. The SK2 band that we used for quantification of the western blots was specific for the SK2 channel as it was absent in the brain homogenates derived from SK2 knockout mice (data not shown).

### Immunohistochemistry

Rats were deeply anesthetized with a Ketamine/Xylazine/Acepromazine mixture and perfused with 4% paraformaldehyde (PFA) in 0.1 M phosphate buffer (n = 4−6). Brains were post-fixed in 4% PFA overnight and processed for immunohistochemistry as previously described [33]. Briefly, brains were cryoprotected in 30% sucrose, frozen in ice-cold isopentane, and stored at −80°C. Thirty micron coronal sections were cut, washed (2×10 min) in 1X phosphate buffered saline (PBS; 137 mM NaCl, 2.7 mM KCl, 4.3 mM Na_2_HPO_4_, 1.47 mM KH_2_PO_4_, pH 7.4) with 0.1% Triton (1X PBS-T), and then incubated in 1X PBS with 0.3% Triton (1X PBS-0.3T) for 20 min. Following incubation in immuno buffer (5% goat serum, 0.3% BSA, 0.3% triton in 1X PBS) for 2 hours at RT, sections were incubated with primary antibodies diluted in immuno buffer. The primary antibodies that were used are as follows: anti-rabbit P-S6 (S240/244)-Alexa fluor-488 conjugate, anti-rabbit P-S6 (S240/244), anti-mouse GFAP (1∶500; Cell Signaling Technology, Boston, MA); anti-mouse Map2, anti-mouse NeuN (1∶1K; Millipore, Temecula, CA); anti-rabbit IBA1 (1∶1K; Wako, Cambridge, MA), and DAPI (1∶50K; 4′,6-diamidino-2-phenylindole; Invitrogen, Carlsbad, CA). Incubation with the primary antibodies was performed for 48 hrs at 4°C on a LabQuake shaker (Barnstead/Thermolyne, Dubuque, IA, USA). Then, the sections were washed in 1X PBS-T (3×10 min) and incubated in Alexa Fluor (594 or 488) labeled goat anti-rabbit or anti-mouse antibodies (1∶10K; Invitrogen, Carlsbad, CA, USA). Sections were mounted in gelatin-covered slides, air-dried and coverslipped with Dako fluorescent mounting medium (Dako, Carpinteria, CA, USA). Staining was visualized using a fluorescent microscope (Nikon Eclipse Ti-S) followed by deconvolution processing for Z stacks. All chemicals were purchased from Sigma Chemical Co. unless otherwise indicated.

### Golgi Staining

Rats were deeply anesthetized with a Ketamine/Xylazine/Acepromazine mixture and perfused with 1X PBS (n = 4−6). Brains were dissected and processed for Golgi staining using the FD Rapid Golgi Stain kit according to manufacturer’s instructions (Neurodigitech, San Diego, CA, USA). Serial coronal sections, 80 µm thick were cut and placed on gelatin-coated slides, stained following the kit’s instructions, dehydrated, and coverslipped using Permount Mounting Medium (Fisher Scientific, Atlanta, GA, USA). Reconstruction of spines and dendrites of CA1 pyramidal neurons was performed using the Neurolucida software (MicroBrightField, Williston, VT, USA). Quantitation of apical dendrite branch points and spine density were performed using the NeuroExplorer software (MicroBrightField). We quantified the number of branch points from the apical dendrites of 4–17 neurons per rat, and evaluated soma area in parallel (n = 4−6 brains/group). To evaluate the number of apical dendritic branch points, we reconstructed dendrites of neurons in which the apical dendrite extended from stratum pyramidale to stratum-lacunosum moleculare. Spine density was determined in 20 µm sections of 3–4 second order dendrites randomly selected per neuron as previously described [34]. Five neurons were randomly selected per brain (n = 4 brains/group); total branches analyzed: Sham+Veh = 59, Sham+Rap = 79, SE+Veh = 77, SE+Rap = 80. ANOVA with Tukey’s *post hoc* test was used to determine significance between groups.

### Statistical Analyses

Analyses for MWM, NOR, vEEG and golgi staining were done blinded to treatment groups. Data analysis was done using the GraphPad Prism software (GraphPad Software, La Jolla, CA, USA). For comparison of two groups student’s t test was used. For multiple comparisons, an ANOVA with Tukey’s *post hoc* test was used. Statistical significance was set at *P<*0.05. All data are shown as mean with standard error of the mean (± SEM).

## Results

### Rapamycin Improved SE-induced Deficits in Hippocampal-dependent Spatial Learning and Memory

Hippocampal-dependent spatial learning and memory deficits have been reported as early as 1–3 weeks following pilocarpine-induced SE [3]. During this time, SE-induced hippocampal mTORC1 hyperactivation occurs in two phases, the first one that peaks at 3 hrs following SE, and a second that peaks at 5 days after SE [9]. In the pilocarpine model of SE, we found an increase in mTORC1 activation that was maximal between days 1–3 after SE, and stabilized at significantly elevated levels between weeks 1–2 in the SE group compared to age-matched controls (Fig. S1). At the two week time point the rats had recovered from the acute effects of SE and were able to perform the behavioral tests, yet mTORC1 activation remained significantly elevated. Thus, we chose this time point for our studies.

**Figure 2:**
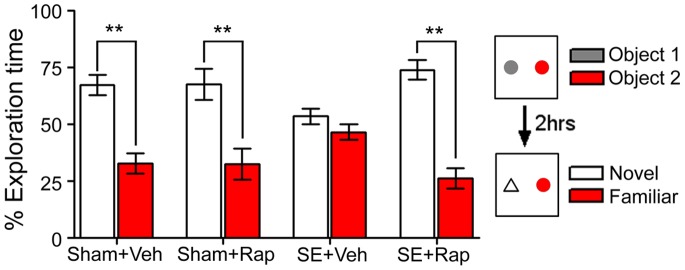
Rapamycin improved recognition memory in rats subjected to SE. The novel object recognition test was used to assess recognition memory in sham and SE rats treated with rapamycin (Rap) or vehicle (Veh). Rats were allowed to explore 2 objects for 5 minutes during the training phase. Two hours later, the rats were re-exposed to a previously explored object (familiar object) and a novel object, and exploration time of each object was evaluated. Sham+Veh and Sham+Rap rats spent significantly more time exploring the novel object than the familiar object. SE+Veh rats spent a similar amount of time exploring both objects. This deficit was reversed by rapamycin in the SE+Rap group. **P<0.001, ANOVA with Tukey’s *post hoc* test; n = 7−11. Error bars = SEM.

Rats were given the mTORC1 inhibitor, rapamycin (6 mg/kg, i.p.) every other day, starting 2 weeks after SE and behavioral testing was performed during weeks 2–3 following SE (Fig. 1A). The control and experimental groups were as follows: 1) sham rats treated with vehicle (Sham+Veh), 2) sham rats treated with rapamycin (Sham+Rap), 3) SE rats treated with vehicle (SE+Veh), and 4) SE rats treated with rapamycin (SE+Rap). We used the Morris water maze (MWM) (Fig. 1) and the novel object recognition (NOR) (Fig. 2) tests to evaluate memory in these groups. Consistent with previous reports, we found that the rats in the SE+Veh group had a longer latency to reach the hidden platform (escape latency) in the training phase of the MWM test compared to Sham+Veh rats [Mixed-design ANOVA, F(3,38) = 6.92, *P<*0.001; Fig. 1B]. In the SE+Rap group, the escape latency was significantly shorter than the SE+Veh group (*P<*0.01), and not significantly different from the Sham+Veh group (*P>*0.05), indicating that rapamycin significantly improved spatial memory acquisition in SE rats. Furthermore, rapamycin had no significant effect on the escape latency of the Sham+Rap group compared to the Sham+Veh group (*P>*0.05), indicating that rapamycin at the dose used in this study (6 mg/kg) did not have an effect on memory acquisition in the sham rats. In the probe trial, SE+Veh rats showed significantly less preference for the quadrant that had housed the hidden platform (target quadrant) compared to Sham+Veh rats (*P<*0.05) (Fig. 1C). In contrast, the SE+Rap rats showed a significant preference for the target quadrant (similar to the sham groups) compared to the SE+Veh rats [Mixed design ANOVA, F(9,114) = 2.75, *P<*0.01; Fig. 1C], indicating improved spatial memory following rapamycin treatments in the SE rats. In the probe trial, rapamycin had no effect on behavior in the Sham+Rap group, demonstrating that rapamycin at this dose had no effect on spatial memory retention in the sham-treated rats.

In the visible platform trial, there were no significant differences in the time to locate a visible platform (*P>*0.05) or in swim speed between the groups (*P>*0.05). Analysis of path length during the MWM revealed that SE+Veh rats took a longer path length than the other groups. There was a main effect of group for path length analysis [F(3,38) = 8.167, *P<*0.001]. The SE+Rap group had a shorter path length than the SE+Veh group and was not different from the Sham+Veh or Sham+Rap groups.

To confirm the rapamycin-mediated rescue in memory in the SE+Rap rats, we used the novel object recognition (NOR) test in a different cohort of rats (Fig. 2). During the training phase rats were allowed to freely explore two sample objects. Rats from all groups spent the same percent of time exploring both objects [F(7,64) = 0.91, *P = *0.50]. Two hours later, rats were re-exposed to one previously explored object (familiar) and one new object (novel). In this test, rats in the Sham+Veh group spent significantly more time exploring the novel object [F(7,64) = 15.09, *P<*0.001], indicating that they remembered the familiar object from the training phase. In contrast, rats from the SE+Veh group spent the same amount of time exploring both the novel and familiar objects, suggesting that these rats did not remember the familiar object. Consistent with the MWM results, rats from the SE+Rap group spent significantly more time exploring the novel object compared to the SE+Veh rats (*P<*0.001). The Sham+Rap and SE+Rap rats performed similar to the Sham+Veh group. Taken together these data indicate that rapamycin treatment significantly improved memory deficits associated with SE.

In addition to memory, we evaluated whether SE-induced mTOR hyperactivity altered locomotor activity and anxiety-like behaviors using the open field test (Fig. S2) (see Methods S1). We found that the total distance the rats traveled in the open field chamber and the mean velocity were significantly increased in the SE+Veh compared to the Sham+Veh group (*P*<0.05), indicating increased locomotor activity in the SE+Veh group. Furthermore, the time spent in the center field of the chamber was significantly increased in the SE+Veh compared to the Sham+Veh group (*P*<0.05), indicating decreased anxiety-like behavior. Rapamycin did not have an effect in the locomotor activity sham or SE rats, but it blocked the preference for the center field in the SE+Rap group, suggesting an effect of rapamycin on the decreased anxiety in the SE group.

Excessive mTOR activation has been associated with aberrant social behavior in rodents [5], [35]. Therefore, in a different cohort of rats we evaluated social behavior using the social interaction test (Fig. S3). We found that SE+Veh rats had altered social interaction compared to Sham+Veh, but rapamycin did not restore normal behavior in the SE rats, and had no effect in the Sham+Rap group.

### Rapamycin had no Effect on Interictal Epileptiform Activity Following SE

**Figure 3:**
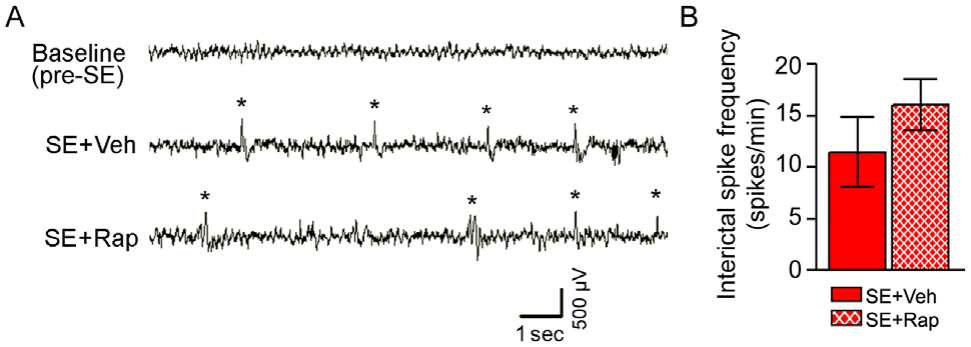
Interictal spike frequency following SE was unaffected by rapamycin. (**A–B**) EEG activity was recorded one week before (baseline) and 2–3 weeks after pilocarpine-induced SE during rapamycin (SE+Rap) or vehicle treatments (SE+Veh). Interictal spikes were quantified from each experimental group. (**A**) Representative EEG traces from cortex show abnormal interictal spike activity (*) in the SE+Veh and SE+Rap groups 2–3 weeks after SE, which is not evident in the baseline EEG trace. (**B**) Quantification of interictal spike frequency showed no differences between SE+Veh and SE+Rap groups. Similar findings were evident in hippocampal EEG traces (data not shown). *P>*0.05, Student’s t test; n = 7−8. Scale bar = 500 µV/sec. Error bars = SEM.

Rapamycin has been shown to reduce seizure frequency and interictal epileptiform activity in acquired and genetic models of epilepsy [9], [28], [32], [36], [37]. For this reason, we evaluated whether rapamycin had an effect on epileptiform activity recorded with vEEG as this could have an effect on behavior. We performed vEEG recordings in a group of rats that did not undergo memory testing since manipulations with electrodes and intermittent EEG recordings might have an effect on the behavioral testing. Video-EEG was recorded one week prior to the induction of SE to determine baseline activity. No abnormal spike activity was evident in the baseline traces prior to pilocarpine. Between weeks 1–2 after SE and prior to the rapamycin or vehicle treatments (pre-treatment) we observed interictal EEG discharges (spikes) in all SE rats. The frequency of interictal activity was not different in rats that were randomly selected for vehicle or rapamycin treatments (pre-vehicle: 12.8±3.2 spikes/min; pre-rapamycin: 11.8±3.0 spikes/min). Two weeks after SE, we recorded vEEG activity during the one week period of vehicle (SE+Veh) or rapamycin (SE+Rap) treatments. We observed interictal spike activity but no electrographic or behavioral seizures in rats that experienced SE (SE+Veh and SE+Rap) (Fig. 3A). There was no significant difference in the frequency of the interictal epileptiform activity in the SE+Rap compared to the SE+Veh rats (Fig. 3B). There was no difference in the spike frequency following single or repeated doses of rapamycin or vehicle (data not shown). In a subset of these rats, hippocampal depth electrodes were implanted, and we found no difference in the frequency of interictal activity in these regions in the SE+Veh compared with SE+Rap groups (data not shown). Thus, rapamycin had no significant effect on interictal epileptiform activity at this time point following SE. Furthermore, these data suggest that the rapamycin-induced improvement in spatial learning and memory in the SE+Rap group was not due to suppression of interictal epileptiform activity.

### Rapamycin Suppressed SE-induced Hyperphosphorylation of mTORC1 Downstream Targets

**Figure 4:**
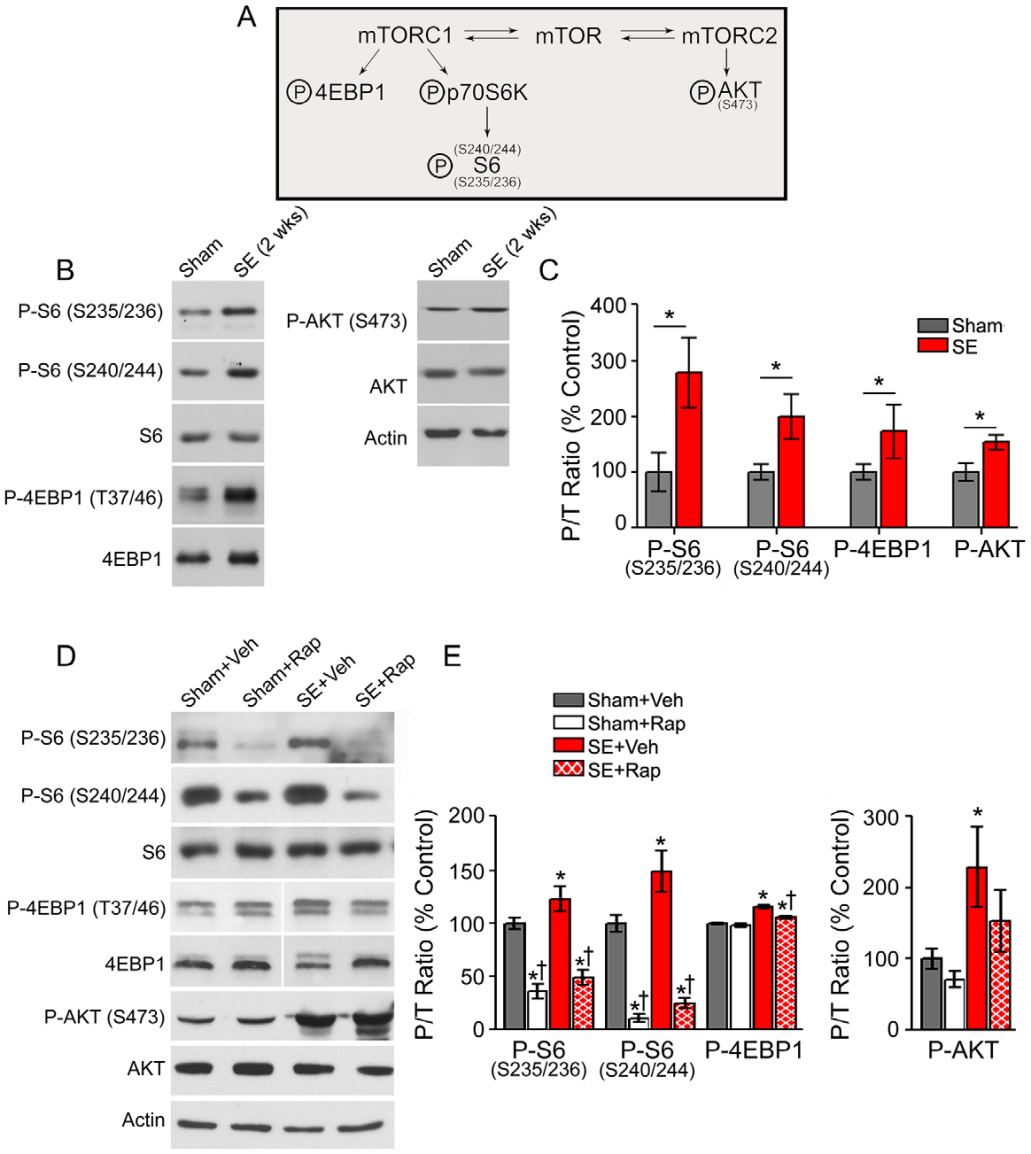
Rapamycin treatment suppressed SE-induced hyperphosphorylation of mTORC1 downstream targets. Western blotting was performed in whole hippocampal homogenates from SE and sham rats 2 weeks after SE (**B**–**C**) and after treatment with rapamycin (Rap) or vehicle (Veh) following the behavioral tests (**D**–**E**). (**A**) Diagram illustrating mTORC1/2 targets evaluated. (**B**) Representative immunoblots from hippocampal proteins derived from sham and SE rats 2 weeks after pilocarpine-induced SE probed for the total and phosphorylated (P-) forms of S6, 4EBP1, AKT and actin are shown. (**C**) Quantitative analysis of the phosphorylated to total protein (P/T) ratio shows a significant increase in P-S6, P-4EBP1 and P-AKT 2 weeks after SE compared to shams. * *P<*0.05, student’s t test; n = 3−5. (**D**) Representative immunoblots from hippocampal proteins derived from Sham+Veh, Sham+Rap, SE+Veh and SE+Rap following behavioral tests, probed for total and P- S6, 4EBP1, AKT, and actin are shown. (**E**) Quantitative analysis of the P/T ratio of immunoreactive bands shows significantly increased levels of P-S6, P-4EBP1, and P-AKT in the SE+Veh group compared to Sham+Veh. Rapamycin suppressed P-S6 levels in the Sham+Rap and SE+Rap groups, and partially reduced P-4EBP1 in the SE+Rap group but not in the Sham+Rap group. P-AKT levels remained elevated despite rapamycin treatment. Note that blots for total and phospho-4EBP1 were run in the same gel but were noncontiguous. * compared to Sham+Veh, *P<*0.05; † compared to SE+Veh, *P<*0.05, ANOVA with Tukey’s *post hoc* test; n = 5−8. Error bars = SEM.

Previous studies have shown that hyperphosphorylation of S6 after an episode of SE is suppressed by rapamycin treatment [8], [9]. However, these previous studies in the kainate and pilocarpine models of SE and epilepsy have only assessed P-S6 at the S235/235 site [8], [9], which can be phosphorylated in an mTOR-independent manner [38]. Thus, following the behavioral tests we used western blotting to confirm the effects of rapamycin on the activation of mTORC1 and 2 downstream targets. Phosphorylation (P) levels of the mTORC1 targets 4EBP1 and S6 at the S240/244 (mTORC1 specific) and S235/236 sites [39], and the mTORC2 target P-AKT (S473) were assessed (Fig. 4A). Western blotting in whole cell hippocampal homogenates revealed a long-lasting dysregulation in the phosphorylation status of these mTOR downstream targets that was evident 2 (Fig. 4B–C) and 3 weeks after SE (Fig. 4D–E). There was a significant increase in the levels of P-S6 at both phospho-regulatory sites in hippocampus at 2 and 3 weeks after SE compared to the shams (*P*<0.05). Rapamycin caused a significant reduction in the levels of P-S6 at both phospho-regulatory sites in the SE and sham groups. Levels of P-4EBP1 were significantly increased in the SE+Veh group compared to Sham+Veh (*P*<0.05). Although, the levels of P-4EBP1 were significantly lower in SE+Rap compared to SE+Veh group (*P<*0.05), these levels remained significantly higher in the SE+Rap group compared with the Sham+Veh group (*P<*0.05). Rapamycin did not significantly alter the levels of P-4EBP1 in the sham group. We found a dysregulation of mTORC2 activity following SE in that there were significantly increased levels of P-AKT (S473 site) in the SE+Veh compared with the Sham+Veh group (*P*<0.05). However, rapamycin had no effect on P-AKT levels (mTORC2 target) in any of the groups (Fig. 4D–E). Thus, the rapamycin effect appeared to be selective for the mTORC1 pathway as expected with short-term treatment [40]. The total protein levels for each phospho-protein analyzed and actin were not significantly different between the groups.

### mTORC1 Hyperactivity Occurred in Neurons and Microglia Following SE

**Figure 5:**
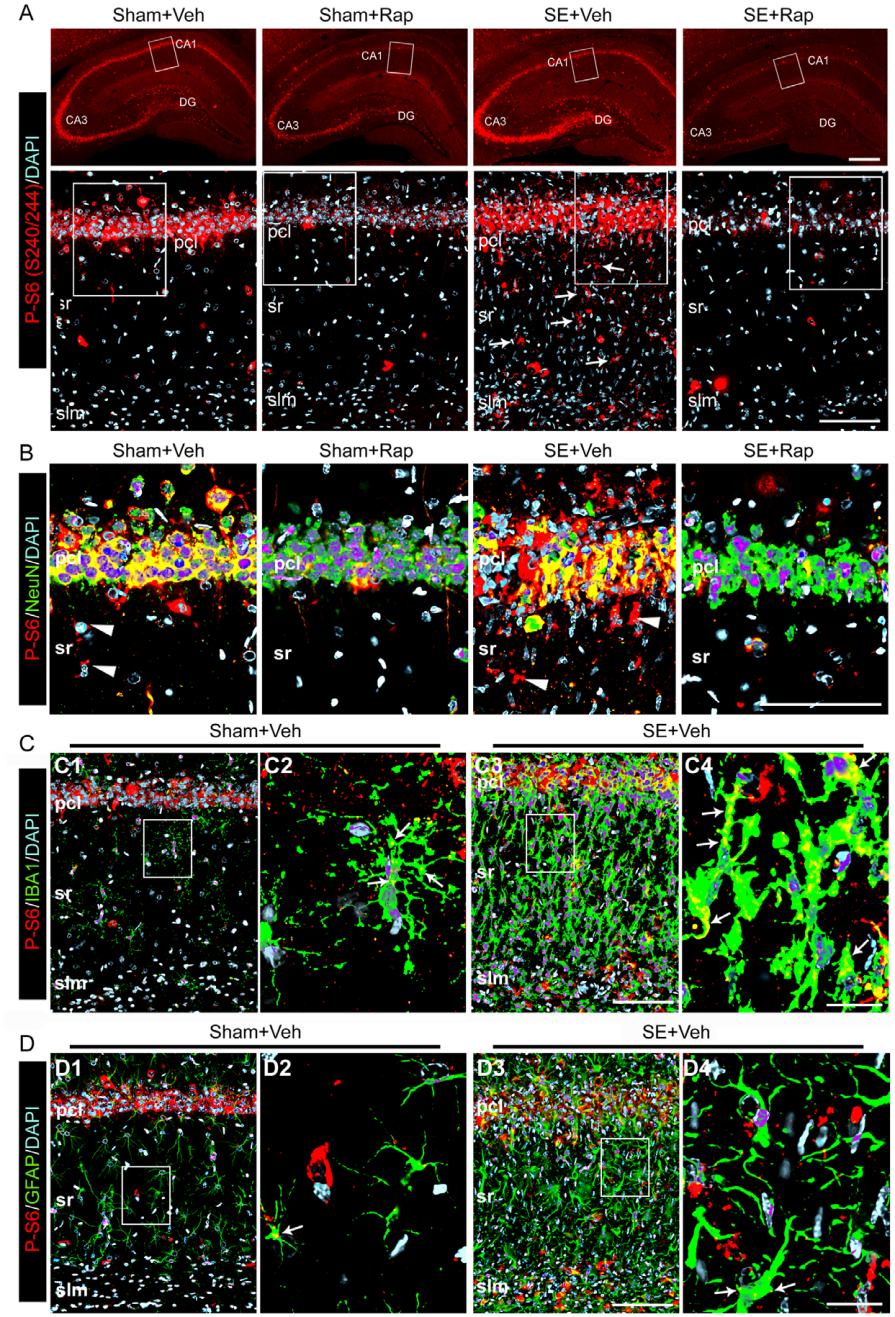
Rapamycin suppressed the SE-induced hyperphosphorylation of the S6 protein in hippocampal neurons and microglia. Immunohistochemistry was performed using antibodies against phosphorylated (P)-S6 (S240/244) on coronal brain sections derived from SE and sham rats after treatment with rapamycin (Rap) or vehicle (Veh) following behavioral tests. (**A**) Representative low (top panels) and high (bottom panels) power images show P-S6 staining (red) in cells localized within the CA1 and DG regions of the Sham+Veh and SE+Veh groups and in glial-appearing cells that is more intense within sr and slm of the SE+Veh group (bottom panel arrows). P-S6 was drastically reduced below basal levels in the rapamycin-treated groups. (**B**) Higher magnification images (boxed on A bottom panels) show co-localization (yellow) of P-S6 staining (red) with NeuN (green) within the CA1 pcl of all groups, but not in some cells localized within sr of the Sham+Veh and SE+Veh groups (arrowhead). (**C–D**) Co-localization (yellow) of P-S6 (red) with IBA1 (green) (arrows) (**C**) and GFAP (**D**) (green) in the Sham+SE was greater than the Sham+Veh group. (**C2, C4, D2, D4**) High magnification images from areas boxed in C1, C3, D1 and D4, respectively. (**B–D**) Deconvoluted maximum projection images are from 21 Z-stacks (0.5 µm steps). Scale bars: **A** top panels: 500 µm; **A** bottom panels, **B**, **C3**, **D3**∶100 µm; **C4**, **D4**∶25 µm; Abbreviations: pcl, pyramidal cell layer; sr, stratum radiatum; slm, stratum lacunosum moleculare; gcl, granule cell layer; DG, dentate gyrus; n = 4−6.

The mTOR pathway has been shown to participate in the regulation of neuronal and microglial properties [10], [17], and astrocytic activation of mTORC1 following SE has been reported [41]. Therefore, we evaluated the localization of the SE-induced aberrant mTOR signaling within the hippocampal formation using immunostaining for P-S6 (S240/244) and colocalization with cellular markers following behavioral assessments. In hippocampi from Sham+Veh rats, P-S6 (S240/244) staining was evident within the CA1–3 pyramidal cell layers (pcl), and in cells scattered throughout the strata radiatum (sr) and lacunosum moleculare (slm), and within the hilus of the dentate gyrus (DG) (Fig. 5A). In the SE+Veh group the P-S6 signal appeared slightly more intense within the pyramidal and granule cell body layers and in cells localized in CA1 sr and slm compared to the Sham+Veh group. Rapamycin reduced the P-S6 signal below basal levels in hippocampi from Sham+Rap and SE+Rap groups. In all groups P-S6 staining co-localized with NeuN (Fig. 5B), indicating neuronal localization of P-S6. There was also co-localization of P-S6 with the microglial marker IBA1 (Fig. 5C), and to a lesser extent with the astrocyte marker GFAP (Fig. 5D) in the Sham+Veh group, which was more pronounced in the SE+Veh group. Due to the low levels of P-S6 staining in the rapamycin-treated groups, we did not find co-localization with the IBA1 or GFAP markers following rapamycin treatments (data not shown). We did not find P-S6 co-localization with oligodendrocytes, Map2, synaptophysin, Gad67, or Neurofilament M in any of the groups (data not shown).

### Rapamycin Suppressed SE-induced Microgliosis in the Hippocampus

**Figure 6:**
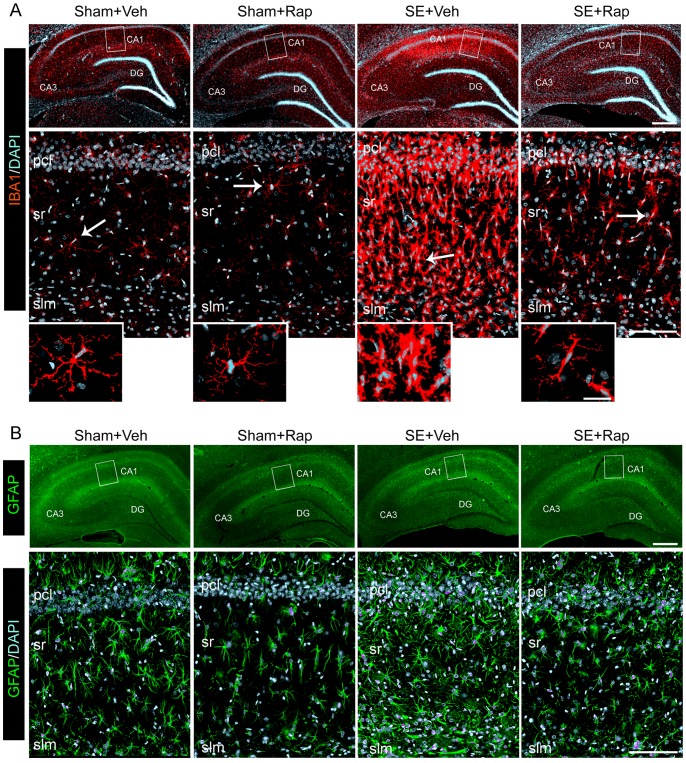
Rapamycin reduced SE-induced hippocampal gliosis. (**A–B**) We used immunohistochemistry to evaluate the distribution of staining for IBA1 (for microglia) (**A**) and GFAP (for astrocytes) (**B**). Immunostaining was performed in hippocampi from SE and sham rats after treatment with rapamycin (Rap) or vehicle (Veh), following behavioral tests. (**A**–**B** top panels) Representative low power images with IBA1 (red) (**A**) and GFAP (green) (**B**) staining within the CA1 and DG regions is shown. DAPI (blue) shows nuclear staining. (**A**–**B** bottom panels) Representative deconvoluted higher magnification projection images of the IBA1 (**A**) and GFAP (**B**) staining within CA1 pcl, sr, and slm. These images show a robust increase in IBA1 (**A**) and GFAP (**B**) staining in the CA1 area of the SE+Veh group that is drastically reduced in the SE+Rap group. Insets in **A** show IBA1-stained microglia with branched processes and small cell bodies in the Sham+Veh, Sham+Rap and SE+Rap groups, which in contrast are hypertrophied and amoeboid shaped in the SE+Veh group (arrows). Scale bars: **A**,**B** top panels: 500 µm; **A**,**B** bottom panels: 100 µm; deconvoluted maximum projection images are from 21 Z-stacks (0.5 µm steps). Abbreviations: pcl, pyramidal cell layer; sr, stratum radiatum; slm, stratum lacunosum moleculare; gcl, granule cell layer; DG, dentate gyrus; n = 6.

An increase in the activation and proliferation of microglia (microgliosis) has been reported in the hippocampus following SE [42], [43]. Thus, given that the SE-induced increase in P-S6 was evident in microglia (Fig. 5C) we evaluated the effects of rapamycin on SE-induced microgliosis in the hippocampus. Immunohistochemistry revealed that the IBA1 staining in hippocampus was dramatically increased in the SE+Veh group compared to Sham+Veh controls. The SE-induced increase in the IBA1 staining was predominantly localized within the CA1 pcl, sr and slm regions (Fig. 6A top panel), and showed a change in the shape of microglia from densely ramified in the Sham+Veh group to hypertrophied and amoeboid in the SE+Veh group (insets). This altered morphology in the SE+Veh group is an indication of microglial activation [44]. Rapamycin drastically reduced the intensity and hypertrophy of the IBA1-stained microglia in the SE+Rap group. The shape of most microglial cells in the SE+Rap group was changed from an amoeboid to a highly branched and elongated morphology (inset). Additionally, we evaluated the effect of rapamycin on the distribution and levels of the GFAP protein present in astrocytes, because abnormal astrocytic activation has been reported in SE [42]. In contrast to the SE-induced increase in IBA1 staining, which was concentrated within area CA1, the GFAP staining was relatively more intense throughout the entire hippocampus in the SE+Veh group compared to the Sham+Veh group and reduced following rapamycin treatment in the SE+Rap group (Fig. 6B). Thus, based on our immunohistochemistry findings, there are basal levels of mTOR activity in neurons and glial cells, and following SE there is hyperactivity of mTOR that involves both cell types. Furthermore, mTOR hyperactivity appears to contribute to microglia activation in hippocampus following SE.

### Rapamycin Reversed SE-induced Alterations in Hippocampal Map2 Levels and Dendritic Branching and Spine Density

**Figure 7:**
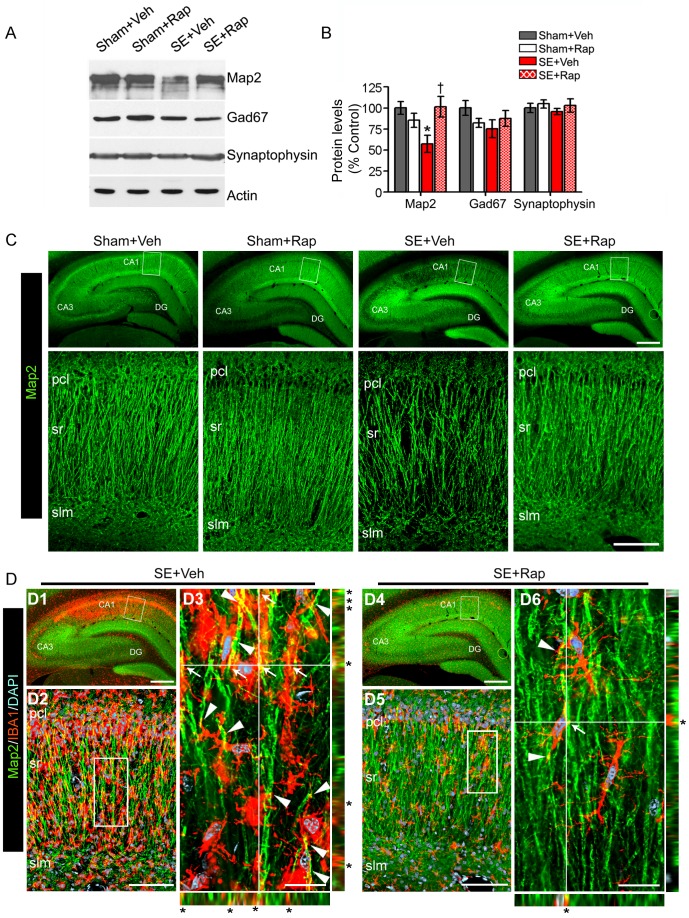
Rapamycin rescues Map2 protein levels in hippocampi from rats subjected to SE. (**A–B**) We used western blotting to measure the levels of Map2, Gad67, synaptophysin and actin in hippocampi from SE and sham rats after treatment with rapamycin (Rap) or vehicle (Veh) following the behavioral tests. (**A**) Representative immunoblots. (**B**) Map2 levels were lower in the SE+Veh compared to the Sham+Veh group and reversed in the SE+Rap group. Levels of Gad67, synaptophysin or actin were not different between the groups. * compared to Sham+Veh, *P<*0.05; † compared to SE+Veh, *P<*0.05, ANOVA with Tukey’s *post hoc* test; n = 5−7. Error bars = SEM. (**C–D**) We used immunohistochemistry to evaluate Map2 distribution. (**C**) Representative low (top panels) and higher (bottom panels) magnification images show prominent Map2 loss within the CA1 sr of the SE+Veh compared with the Sham+Veh group that was reversed in the SE+Rap group. (**D**) Co-immunostaining with Map2 and IBA1 antibodies show a correlation in their localization in the SE+Veh group that is diminished in the SE+Rap group. (**D2–3, D5–6**) The images with corresponding XZ and YZ projections show microglia processes (red) intertwined with (arrowheads) and touching (arrows/asterisks) (yellow) the Map2-labeled dendrites (green) in the SE+Veh (**D3**) and SE+Rap (**D6**) groups. Deconvoluted maximum projection images: **C, D2, D5∶**21 Z-stacks (0.5 µm steps); **D3, D6∶**31 Z-stacks (0.1 µm steps). Scale bars: **C** top panels, **D1**, **D4**∶500 µm; **C** bottom panels, **D2**, **D5**∶100 µm; **D3**, **D6**∶20 µm. Abbreviations: pcl, pyramidal cell layer; sr, stratum radiatum; slm, stratum lacunosum moleculare; DG, dentate gyrus; n = 6.

We evaluated whether rapamycin reversed the SE-induced changes in hippocampal dendrites in parallel with the observed behavioral rescue. Structural alterations and instability in hippocampal dendrites have been described following chronic seizure activity [20], [21], [22], [45]; however, the mechanism underlying these changes is unclear and whether these changes are evident early following SE before full-blown epilepsy has evolved is unknown. Using western blotting with antibodies against the dendritic marker Map2, we found a significant reduction in the hippocampal Map2 protein levels in the SE+Veh compared to the Sham+Veh group (*P<*0.05) that were rescued to basal levels in the SE+Rap group (Fig. 7A–B). Rapamycin did not alter Map2 protein levels in the Sham+Rap group compared to controls (*P>*0.5). At this time point, the protein levels of the presynaptic markers Gad67 and synaptophysin were not significantly different between the groups (*P>*0.05). Thus, we focused on immunohistochemistry using the Map2 antibody. We found a dramatic reduction in Map2 staining in the SE+Veh compared to the Sham+Veh group, which was most notable within the CA1 sr and slm (Fig. 7C). Map2-stained dendrites within CA1 sr were visibly disorganized and less dense in hippocampi from SE+Veh compared to the Sham+Veh group. In hippocampi from SE+Rap rats, Map2-stained dendrites appeared more organized and dense compared to those from the SE+Veh group. Furthermore, the localization of the Map2 loss corresponded to regions of microgliosis in area CA1 from the SE+Veh group (Fig. 7D) (see microglia image from Fig. 5A). Notably, IBA1-stained microglia were intertwined with (arrowheads) and touching (arrows and asterisks on YZ and XZ axis) the Map2-labeled dendrites in area CA1 from the SE+Veh group. This effect was dramatically reduced in the SE+Rap group (Fig. 7D) and was similar to the Sham+Veh group (Fig. S5).

**Figure 8:**
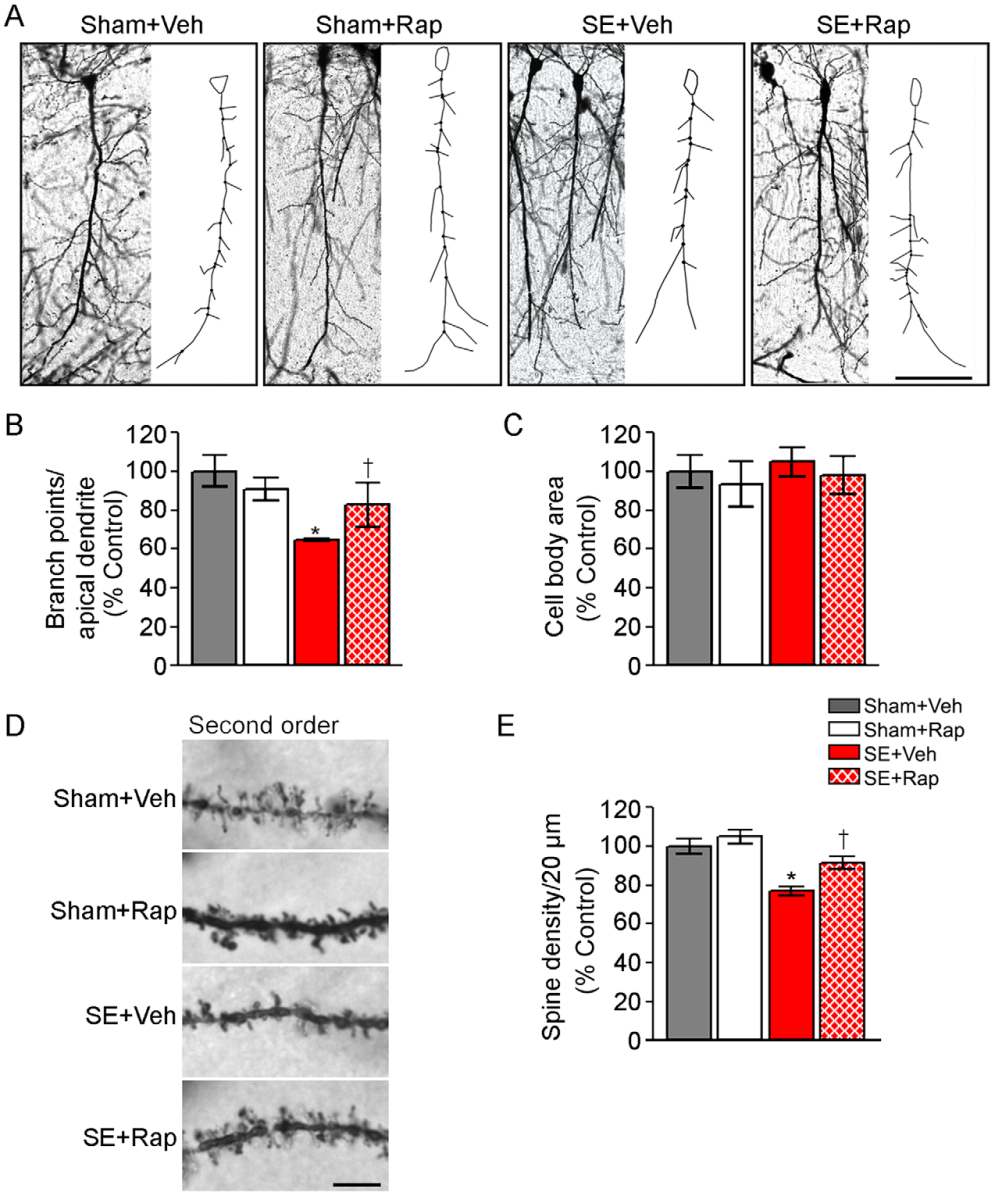
Rapamycin improved CA1 dendritic arborization and spine density in hippocampi from rats subjected to SE. We used golgi staining followed by neurolucida reconstructions to determine dendritic branching and spine density in CA1 cells from SE and sham rats after treatment with rapamycin (Rap) or vehicle (Veh) following the behavioral tests. (**A**) Representative images of golgi-stained CA1 neurons and their respective reconstructions showing the branch points from the apical dendrites. (**B**) Branch point analysis normalized to percent of Sham+Veh (% control) shows a significant decrease in the apical dendrite branch points in the SE+Veh compared to the Sham+Veh group that is reversed in the SE+Rap group; n = 4−6 brains/group from which 4–17 cells/brain were analyzed. (**C**) Analysis of cell body area shows no differences between the groups. (**D**) Representative images of 20 µm sections of second order dendritic branches and spines; n = 59−80 branches/group. (**E**) Quantitative analysis of spine density in secondary dendritic branch structures shows a significant decrease in spine density from the SE+Veh compared to the Sham+Veh group that is reversed in the SE+Rap group. Scale bar = **A**: 100µm; **D**: 5µm. * compared to Sham+Veh, *P<*0.05; † compared to SE+Veh, *P<*0.05, ANOVA with Tukey’s *post hoc* test. Error bars = SEM.

To determine whether dendritic arborization and spine density of CA1 cells improved with rapamycin treatment following SE we performed dendritic reconstructions after golgi staining and quantified these changes using neurolucida (Fig. 8). We found a significant reduction (∼30%) in the number of branch points from the apical dendrites of the CA1 neurons from the SE+Veh compared to those from the Sham+Veh group (*P*<0.05) (Fig. 8A–B). This effect was reversed with rapamycin such that the number of branch points was significantly increased in the SE+Rap compared to the SE+Veh group (*P*<0.05), while no significant changes were observed in the Sham+Rap relative to the Sham+Veh group. The cell body area was unaffected by SE or rapamycin treatments (Fig. 8C). The spine density in dendritic branches emerging from the apical dendrite (second order branches) was assessed (Fig. 8D–E). We found a significant decrease (∼20%) in the spine density in the SE+Veh compared to the Sham+Veh group (*P*<0.05) that was reversed in the SE+Rap group (SE+Veh vs. SE+Rap, *P*<0.05). Rapamycin did not significantly alter the spine density in the Sham+Rap compared to Sham+Veh group. Spine density in higher level dendritic branches was not determined due to extensive dendritic damage in the SE+Veh group.

### Rapamycin Reversed SE-induced Alterations in Hippocampal Dendritic Ion Channels

**Figure 9:**
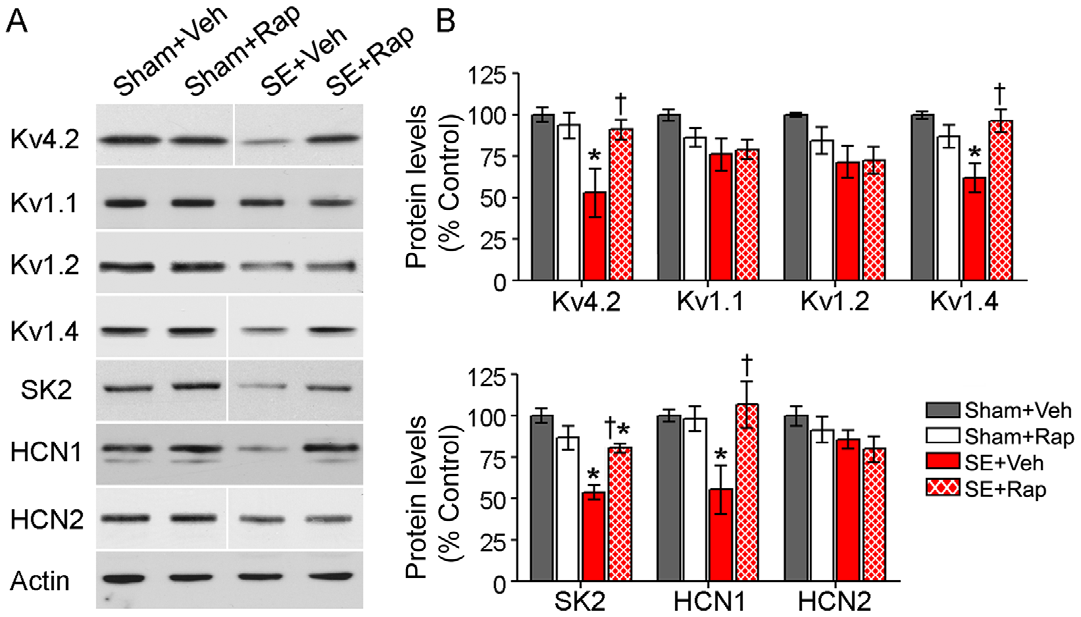
Rapamycin restored protein levels of dendritic ion channels in hippocampi from rats subjected to SE. (**A–B**) We used western blotting to measure the protein levels of several ion channels in hippocampi from SE and sham rats after treatment with rapamycin (Rap) or vehicle (Veh) following the behavioral tests. (**A**) Representative western blots probed with the antibodies against Kv4.2, Kv1.1, Kv1.2, Kv1.4, SK2, HCN1, HCN2 channels and actin are shown. (**B**) Analysis of immunoreactive bands revealed significantly lower levels of Kv4.2, Kv1.4, SK2, and HCN1 in the SE+Veh compared with the Sham+Veh group. In the SE+Rap group, rapamycin rescued basal levels of Kv4.2, Kv1.4, and HCN1 channels with a partial rescue of SK2. Levels of Kv1.1, Kv1.2 or HCN2 were not changed in the SE group and rapamycin treatment had no effect on these channels. Note that blots for Kv4.2, Kv1.4, SK2 and HCN2 were run in the same gel but were noncontiguous. * compared to Sham+Veh, *P<*0.05; † compared to SE+Veh, *P<*0.05, ANOVA with Tukey’s *post hoc* test; n = 5−8; SK2: n = 3−4. Error bars = SEM.

Alterations in a number of ion channels localized to hippocampus have been reported following SE [24], [25], [26], [31], [46]. Thus, given the rapamycin-mediated improvement in the CA1 dendritic structure in the rats subjected to SE (Figs. 7–8) and that mTOR signaling has been shown to regulate expression of dendritic voltage-gated potassium (Kv) channels [12], [13], we evaluated the effects of rapamycin on SE-mediated changes in several of the ion channels localized to hippocampus (Fig. 9). Western blotting confirmed the previously described SE-induced reductions in Kv4.2, Kv1.4, SK2, and HCN1 in hippocampus two and three weeks following SE (Sham+Veh vs. SE+Veh, *P<*0.05) (Fig. S4D and Fig. 9, respectively). Rapamycin treatment significantly increased the protein levels of these channels in the SE+Rap compared to the SE+Veh group (*P<*0.05). In the SE+Rap group, protein levels of Kv4.2, Kv1.4, and HCN1 channels were not significantly different from those of the Sham+Veh group (*P>*0.05). Although, the protein levels of the SK2 channel were significantly higher in SE+Rap compared to SE+Veh group (*P<*0.05), these levels remained significantly lower in the SE+Rap group compared with the Sham+Veh group (*P<*0.05), suggesting a partial rescue of SK2 protein levels. Levels of Kv1.1, Kv1.2, and HCN2 were unaffected by the rapamycin treatments. Thus, in parallel with rescue of dendritic structure, rapamycin also rescued molecular alterations in hippocampal dendrites following SE involving ion channels known to play a key role in synaptic plasticity and learning and memory [29], [47], [48], [49].

## Discussion

A growing body of evidence implicates aberrant mTORC1 signaling in the neuropathology of many neurological disorders, in particular genetic disorders associated with cognitive and social deficits and epilepsy [5], [6], [7], [11], [50]. Furthermore, cognitive impairments are a common comorbidity in acquired epilepsy that follows SE [2], [3], [4]. Previous studies have shown aberrant mTORC1 activation following SE in rodent models [8], [9]. In our studies we build upon this previous work, extending these findings to show for the first time a link between mTOR dysregulation and hippocampal-dependent learning and memory deficits associated with SE. Rats that experienced SE and subsequently were treated with rapamycin 2 weeks later, performed significantly better than vehicle-treated SE rats in two hippocampal-dependent memory tasks, MWM and NOR (Figs. 1–2). Additionally, we describe mTOR-dependent activation of microglial cells in association with structural and molecular dendritic alterations following SE that are reversed with rapamycin (Figs. 4, 5, 6, 7). Here we show that at the molecular level, the SE-induced increase in mTORC1 activation through P-S6 occurs in reactive microglial cells and neurons (Fig. 5), and that inhibition of mTORC1 attenuated the SE-induced microgliosis (Fig. 6), improved dendritic arborization (Figs. 7–8), and restored protein levels of hippocampal ion channels (Fig. 9). In parallel, we found that SE-induced microgliosis correlated with dendritic damage in the area CA1 of hippocampus (Fig. 7). Taken together, our findings implicate aberrant mTORC1 activation as a candidate mechanism involved in hippocampal-dependent cognitive deficits and dendritic pathology associated with SE.

The use of genetic models and pharmacological tools to manipulate mTORC1 activation implicate this pathway as an essential player in the modulation of memory [10], [11], [51]. Transgenic rodents with loss of at least one allele of the upstream mTORC1 negative regulators the phosphatase and tensin homolog (Pten) or the tuberous sclerosis complex 1 and 2 (TSC1/2) proteins are characterized by mTOR pathway hyperactivity, and social and memory deficits, which are reversed with rapamycin [5], [6], [7], [32]. These studies suggest that up-regulation of mTOR signaling alters memory processing, strongly supporting a critical role for the mTOR pathway in memory modulation. For this reason we evaluated whether the SE-induced mTOR hyperactivation [8], [9] contributes to the memory deficits associated with SE and found that rapamycin rescued SE-induced hippocampal-dependent spatial learning and memory deficits (Figs. 1–2). Our findings implicate mTORC1 dysregulation in the memory dysfunction that develops early after SE.

The effects of rapamycin in behavioral deficits in several developmental epilepsy models have been evaluated [50], [52]. A recent study shows that rapamycin improves learning and memory in a rat model of infantile spams [52], further supporting a role for mTORC1 dysregulation in the cognitive deficits associated with epilepsy. In a model of early-life seizures induced by acute hypoxia in neonatal rodents rapamycin rescued aberrant social behavior [50]. However, in our study SE also was associated with altered social interaction, but rapamycin did not rescue the abnormal social behavior (Fig. S3), suggesting different mechanisms for the altered social behavior in these models. Furthermore, a recent study showed that aggressive behavior in pilocarpine-treated epileptic rats is reversed by rapamycin [53].

We found that rapamycin had no significant effect in the performance of sham-treated rats in the behavioral tests (Figs. 1–2), consistent with other studies. For instance, rapamycin rescued deficits in hippocampal-dependent spatial learning tasks in a TSC2 (+/−) mouse model but had no effect on the performance of the wild type (WT) mice on those tests [6]. Several other studies have reported that rapamycin impairs learning and memory in normal rats and WT mice, but much higher doses (e.g. 150 mg/kg) or direct CNS infusion (e.g. 5–1000 ng) of rapamycin were required for this effect [6], [54], [55]. Taken together these studies indicate that a higher level of mTORC1 inhibition is required to disrupt learning and memory in normal rodents, thus explaining the lack of effect of rapamycin on the behavioral tests in the sham rats described here.

Previous findings showing that rapamycin suppresses spontaneous seizures in epileptic rodents [9], [28], [32], [37] would have led us to predict a reduction in epileptiform activity in the SE group treated with rapamycin; however, in our studies acute rapamycin treatment given weeks 2–3 following SE had no effect on epileptiform activity during the period of our studies (Fig. 3). Furthermore, we cannot exclude the possibility that seizures might have occurred at times when the rats were not being monitored. In line with our findings, recent studies showed that short-term rapamycin treatments have limited anticonvulsive effects in rodent models of acute seizures [56], [57]. In addition, Buckmaster and Lew (2011) showed that long-term rapamycin treatment beginning one day after SE induction was not sufficient to reduce seizure frequency in the pilocarpine model of epilepsy [58]. The discrepancies between these studies may be attributed to the timing of rapamycin administration (i.e. pre- vs. post-SE induction), length of rapamycin treatment (acute vs. long-term), different rodent species, and the epilepsy models used. Interestingly, it has been shown that rapamycin did not alter interictal epileptiform activity in a rat model of infantile spasms [52]. Similarly, some anticonvulsants are suboptimal in reducing interictal spikes [59].

Our finding that SE-induced altered mTOR signaling occurs in both neurons and microglia suggests that mTOR effectors are activated in diverse cells types following SE and may thereby change neuronal and glial properties. In parallel with a reduction in P-S6 staining (Figs. 4–5), there was a decrease in microgliosis in the CA1 region of the SE+Rap group when compared to the SE+Veh group (Fig. 6). In a separate cohort of rats, we found that these alterations were already evident 2 weeks after SE, suggesting that rapamycin reversed the changes (Fig. S4). These data also suggest that SE-induced dendritic damage is mTOR-dependent and that microgliosis may play a role in this process. Indeed, it has been shown that lipopolysaccharide- and cytokine-induced pro-inflammatory activation of microglia is mTOR-dependent [17], [18] and microgliosis is suppressed with rapamycin [17], [18], [60], [61]. Under physiological conditions microglia play critical roles in synaptic pruning during development [62] and in mature synaptic connections [63]. Furthermore, prolonged microglia-synapse contacts induced by ischemia are followed by a large loss of synapses [63], and lipopolysaccharide-induced microgliosis results in loss of spine density and dendritic branching [64]. Based on these studies we speculate that the presence of reactive microglia within CA1 following SE may contribute to the significant loss of dendrites and spines in this region (Fig. 8). Hippocampal microgliosis has been widely reported in humans and animal models of epilepsy [43] and existing evidence suggests that microgliosis in the hippocampus contributes to cognitive deficits [65].

Altered cognitive behavior that occurs in association with epilepsy is often associated with structural [20], [21], [23] and molecular dendritic abnormalities [24], [25]. Thus, it is possible that the rapamycin-mediated reduction in the SE-induced microgliosis promotes dendritic repair and memory improvement in SE rats. In fact, rapamycin has been shown to reduce microgliosis and in parallel improve functional recovery in several neurobehavioral tasks following traumatic brain injury in rodents [60]. Astrogliosis also may contribute to CA1 dendritic damage following SE; however the SE-induced astrogliosis was evident throughout the hippocampus (Fig. 6) and microgliosis was concentrated in areas of high Map2 loss. While the finding that rapamycin reduced SE-induced gliosis in the hippocampus was unexpected, it was not surprising, as rapamycin is a potent immunosuppressant [61]. This finding is important because it opens up the possibility that the beneficial effect of rapamycin on SE-induced phenotypes is at least in part due to an effect on glial responses in the hippocampus. Neuronal death associated with seizures also may play a role in the altered cognitive behavior [66]. Previous studies have shown that rapamycin treatment did not reverse the SE-induced hippocampal cell loss when assessed early or late following SE [8], [9]. Thus, the rapamycin-dependent reduction in SE-induced microgliosis and the improvement of dendritic arborization after SE may at least in part compensate for the loss of neurons to improve spatial memory. Future studies are needed to further evaluate these possible mechanisms following SE.

In neurons, mTORC1 regulates dendritic architecture [15], [16] and modulates dendritic synthesis of Map2 [14]. Association of Map2 with microtubules, kinases, and other proteins is critical for dendritic stability, protein trafficking, neurite initiation, and local signal transduction in neurons [67], [68]. For this reason we speculate that by modulating Map2 levels or other proteins such as ion channels, rapamycin may promote dendritic structural and functional integrity following SE. This is particularly important because studies *in vitro* have shown mTORC1-dependent modulation of dendritic surface expression and protein synthesis of Kv1.1 and Kv4.2 channels, respectively [12], [13]. Our finding that rapamycin restored basal levels of Kv4.2 and HCN1 channels and partially rescued SK2 levels in hippocampus of rats subjected to SE (Fig. 9) suggests that mTOR signaling may contribute to the translational regulation of these channels or associated regulatory proteins in neurons. Under physiological conditions the currents generated by Kv4.2, HCN1, and SK2 contribute to shaping the response to synaptic inputs, modulating neuronal excitability, and learning and memory [47], [48], [49]. Dysregulation of these channels following SE is thought to alter post-synaptic responses through the modulation of the respective underlying currents [24], [25]. This in turn may alter limbic circuitry and thereby contribute to SE-mediated hippocampal-dependent learning and memory deficits. Indeed, genetic alterations in the levels of these channels cause aberrant synaptic plasticity and learning and memory [29], [47], [48], [49]. Based on these studies, we propose that the rapamycin-mediated dendritic repair and reversal of ion channel abnormalities contributes to improvement in the SE-induced memory deficits. In addition, to the dendritic channels, we found a rescue in Kv1.4 protein levels. Because Kv1.4 channel localization also has been described presynaptically within the hippocampus [69], our finding opens up the possibility of an effect of SE-induced mTORC1 dysregulation presynaptically. Additional studies are required to dissect out the specific effects of the SE-induced mTOR dysregulation in neuronal vs. glial cells, and the specific roles of these cell types may play in the associated memory and dendritic dysregulation.

In conclusion, our findings suggest that aberrant mTORC1 signaling contributes to SE-induced hippocampal-dependent spatial learning and memory deficits. While additional studies are required to determine direct molecular targets of this pathway within neurons and microglia, our findings indicate that following SE mTORC1 dysregulation contributes to memory deficits, microgliosis, and dendritic abnormalities that are associated with SE. Given that suppression of mTORC1 signaling improves cognition in humans [70], our findings suggest that the mTORC1 pathway may be a potential therapeutic target for cognitive dysfunction associated with epilepsy.

## Supporting Information

Figure S1 **Pilocarpine-induced SE promotes a long-lasting dysregulation in the phosphorylation status of mTOR downstream targets.** (**A**) Representative immunoblots from total hippocampal homogenates probed with antibodies against the total and phosphorylated (P) forms of S6 [Serine (S) 240/244, S235/236], 4EBP1 [Threonine (T) 37/46], AKT (S473), and actin at 1, 3, and 7 days after SE and sham treatments (controls) are shown. (**B–E**) Quantitative analysis of the phosphorylated to total protein (P/T) ratio normalized to percent of controls (% control). (**B–D**) A significant and long-lasting hyperphosphorylation of S6 (S240/244) (**B**), S6 (S235/236) (**C**) and 4EBP1 (T37/46) (**D**) was evident between 1–7 days after SE compared to age-matched shams. SE-induced phosphorylation of S6 and 4EBP1 peaked between 1–3 days after SE. (**E**) The phosphorylation of AKT (S473) was transiently decreased between days 1–3 after SE and was not significantly different between sham and SE on day seven after SE. * *P*<0.05 by t test. Error bars = SEM, n = 3−8.
(TIF)

Figure S2 **SE-induced abnormal behaviors in the open field test were partially rescued with rapamycin.** We used the open field test to assess locomotor activity and preference for the inner portion of the open field (anxiety measure) in SE and sham rats during treatment with rapamycin (Rap) or vehicle (Veh). (**A**–**B**) There was a significant change in the total distance travelled and the speed of locomotion in the open field test (*P*<0.05). The total distance travelled (**A**) and the mean velocity (**B**) were significantly increased in the SE+Veh compared to the Sham+Veh group and rapamycin had no effect on the activity levels in the sham and SE groups. (**C**) The SE+Veh group spent significantly more time in the inner portion of the open field compared with the Sham+Veh group, while rapamycin treatment blocked this effect in the SE+Rap group. (**D**) Similarly, the SE+Veh group entered the center more frequently than the Sham+Veh and Sham+Rap groups. * compared to Sham+Veh, *P*<0.05; † compared to SE+Veh, *P<0.05,* ANOVA with Tukey’s *post hoc* test. Error bars = SEM, n = 7−8.
(TIF)

Figure S3 **Rapamycin did not reverse the aberrant social behavior in rats subjected to SE.** We used a social interaction test to assess social behavior in SE and sham rats during treatment with rapamycin (Rap) or vehicle (Veh). (**A**) There were no differences in the frequency of active social investigation between the groups. (**B**) There was a significant decrease in time spent in active social behavior in the SE+Veh group compared to the Sham+Veh group that was not reversed in the SE+Rap group. * compared to Sham+Veh, *P*<0.05, ANOVA with Tukey’s *post hoc* test. Error bars = SEM, n = 7−8.
(TIF)

Figure S4 **Reactive microglia expressed P-S6 and microgliosis correlated with Map2 loss in hippocampus from rats 2 weeks following SE.** We used immunohistochemistry to evaluate P-S6, IBA1, and Map2 distribution in hippocampus from sham and SE rats two weeks after SE. (**A**) Representative low (**A1**, **A3**) and high (**A2**, **A4**) power images show IBA1 (green) and P-S6 (S240/244) (red) staining in the CA1 area of sham and SE (2 wks) rats. There was relatively greater P-S6 signal co-localized with IBA1 (yellow) (arrows) in stratum radiatum of the SE (2 wks) compared to the sham group, n = 3. (**B**) Representative low power images show representative hippocampal sections from sham and SE groups stained with antibodies against Map2 (green), IBA1 (red) and Dapi (blue). There is loss of Map2 staining and increased IBA1 signal in the CA1 region of the SE (2 wks) group compared to the sham group, n = 3. (**C**) High magnification images show loss of Map2 staining and Map2-labeled dendrites in the CA1 region of the SE (2 wks) compared with the sham group, n = 3. (**D**) Western blot analysis revealed significantly lower levels of Kv4.2 and HCN1 channel protein in the SE (2 wks) group compared with the sham group, n = 4−5. The deconvoluted maximum projection images shown in **A1** and **A3** are from 21 Z-stacks (0.5 µm steps), and those shown in **A2** and **A4** are from 13 Z-stacks (0.25 µm steps). Scale bars = **A3,C**: 100 µm; **A4**∶25 µm; **B**: 500 µm. Abbreviations: pcl, pyramidal cell layer; sr, stratum radiatum; slm, stratum lacunosum moleculare; DG, dentate gyrus; n = 3−4.
(TIF)

Figure S5 **Rapamycin did not alter the organization and distribution of Map2 or IBA1 staining in the hippocampus of sham rats.** We used immunohistochemistry to evaluate Map2 (green) and IBA1 (red) distribution in hippocampi from Sham+Veh (**A1–3**), Sham+Rap (**A4–6**) and SE+Veh rats (**B**). Dapi (blue) was used to stain cellular nuclei. (**A**) Representative low (**A1, A4**) and high (**A2, A5**) power images (boxed in top panels) are shown for the Sham+Veh and Sham+Rap groups, respectively. (**A3, A6**) Higher magnification images show microglial processes (red) intertwined with and touching (arrows and arrowheads) (yellow) the Map2-labeled dendrites (green) in these groups (area boxed in **A2** and **A5**). This effect is evident in the XZ and YZ projections (arrows and asterisks). (**B**) Representative images from the SE+Veh group show a different CA1 region from the same hippocampus that is shown in figure 7D. (**B7**) The images show microglial processes intertwined with and touching the Map2-labeled dendrites in the SE+Veh group that is evident in the XZ and YZ projections (arrows and asterisks). The deconvoluted maximum projection images shown in **A2**, **A5**, **B2**, **B5** and **B6** are from 21 Z-stacks (0.5 µm steps), and those shown in **A3**, **A6** and **B7** are from 31 Z-stacks (0.1 µm steps). Scale bars: **A4**, **B5**∶500 µm; **A5**, **B6**∶100 µm; **A6**, **B7**∶25 µm. Abbreviations: pcl, pyramidal cell layer; sr, stratum radiatum; slm, stratum lacunosum moleculare; DG, dentate gyrus; n = 4−6.
(TIF)

Methods S1 **Supporting Methods.**
(DOCX)
