# Supplementary figures and images for: Rapamycin Reverses Status Epilepticus-Induced Memory Deficits and Dendritic Damage
Source: PLoS One. 2013 Mar 11;8(3):e57808. doi: 10.1371/journal.pone.0057808 (PMC3594232; doi:10.1371/journal.pone.0057808)

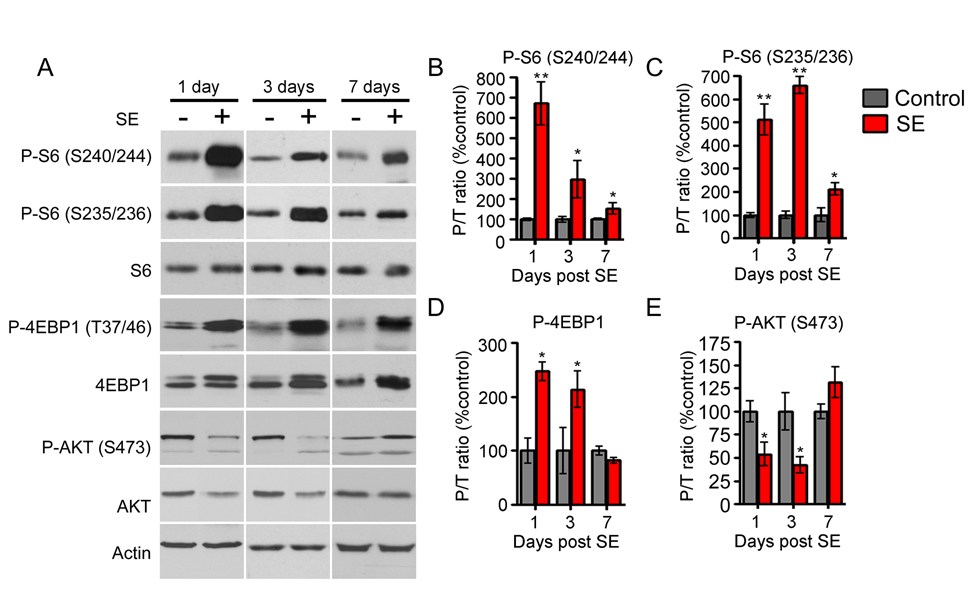

Supplement: Figure S1 — Pilocarpine-induced SE promotes a long-lasting dysregulation in the phosphorylation status of mTOR downstream targets. (A) Representative immunoblots from total hippocampal homogenates probed with antibodies against the total and phosphorylated (P) forms of S6 [Serine (S) 240/244, S235/236], 4EBP1 [Threonine (T) 37/46], AKT (S473), and actin at 1, 3, and 7 days after SE and sham treatments (controls) are shown. (B–E) Quantitative analysis of the phosphorylated to total protein (P/T) ratio normalized to percent of controls (% control). (B–D) A significant and long-lasting hyperphosphorylation of S6 (S240/244) (B), S6 (S235/236) (C) and 4EBP1 (T37/46) (D) was evident between 1–7 days after SE compared to age-matched shams. SE-induced phosphorylation of S6 and 4EBP1 peaked between 1–3 days after SE. (E) The phosphorylation of AKT (S473) was transiently decreased between days 1–3 after SE and was not significantly different between sham and SE on day seven after SE. * P<0.05 by t test. Error bars = SEM, n = 3−8. (TIF) [file pone.0057808.s001.tif]

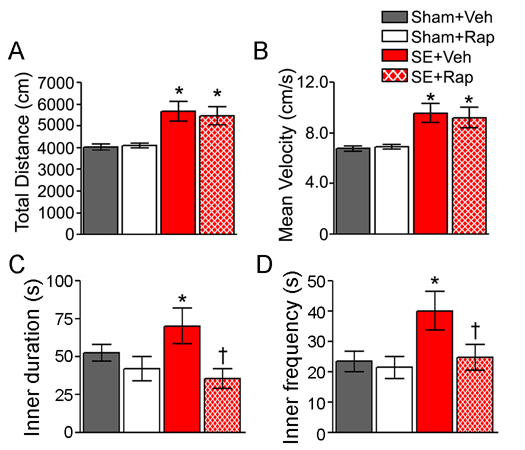

Supplement: Figure S2 — SE-induced abnormal behaviors in the open field test were partially rescued with rapamycin. We used the open field test to assess locomotor activity and preference for the inner portion of the open field (anxiety measure) in SE and sham rats during treatment with rapamycin (Rap) or vehicle (Veh). (A–B) There was a significant change in the total distance travelled and the speed of locomotion in the open field test (P<0.05). The total distance travelled (A) and the mean velocity (B) were significantly increased in the SE+Veh compared to the Sham+Veh group and rapamycin had no effect on the activity levels in the sham and SE groups. (C) The SE+Veh group spent significantly more time in the inner portion of the open field compared with the Sham+Veh group, while rapamycin treatment blocked this effect in the SE+Rap group. (D) Similarly, the SE+Veh group entered the center more frequently than the Sham+Veh and Sham+Rap groups. * compared to Sham+Veh, P<0.05; † compared to SE+Veh, P<0.05, ANOVA with Tukey’s post hoc test. Error bars = SEM, n = 7−8. (TIF) [file pone.0057808.s002.tif]

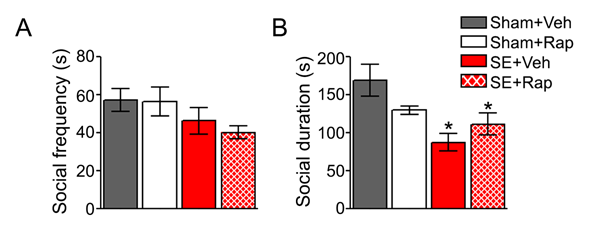

Supplement: Figure S3 — Rapamycin did not reverse the aberrant social behavior in rats subjected to SE. We used a social interaction test to assess social behavior in SE and sham rats during treatment with rapamycin (Rap) or vehicle (Veh). (A) There were no differences in the frequency of active social investigation between the groups. (B) There was a significant decrease in time spent in active social behavior in the SE+Veh group compared to the Sham+Veh group that was not reversed in the SE+Rap group. * compared to Sham+Veh, P<0.05, ANOVA with Tukey’s post hoc test. Error bars = SEM, n = 7−8. (TIF) [file pone.0057808.s003.tif]

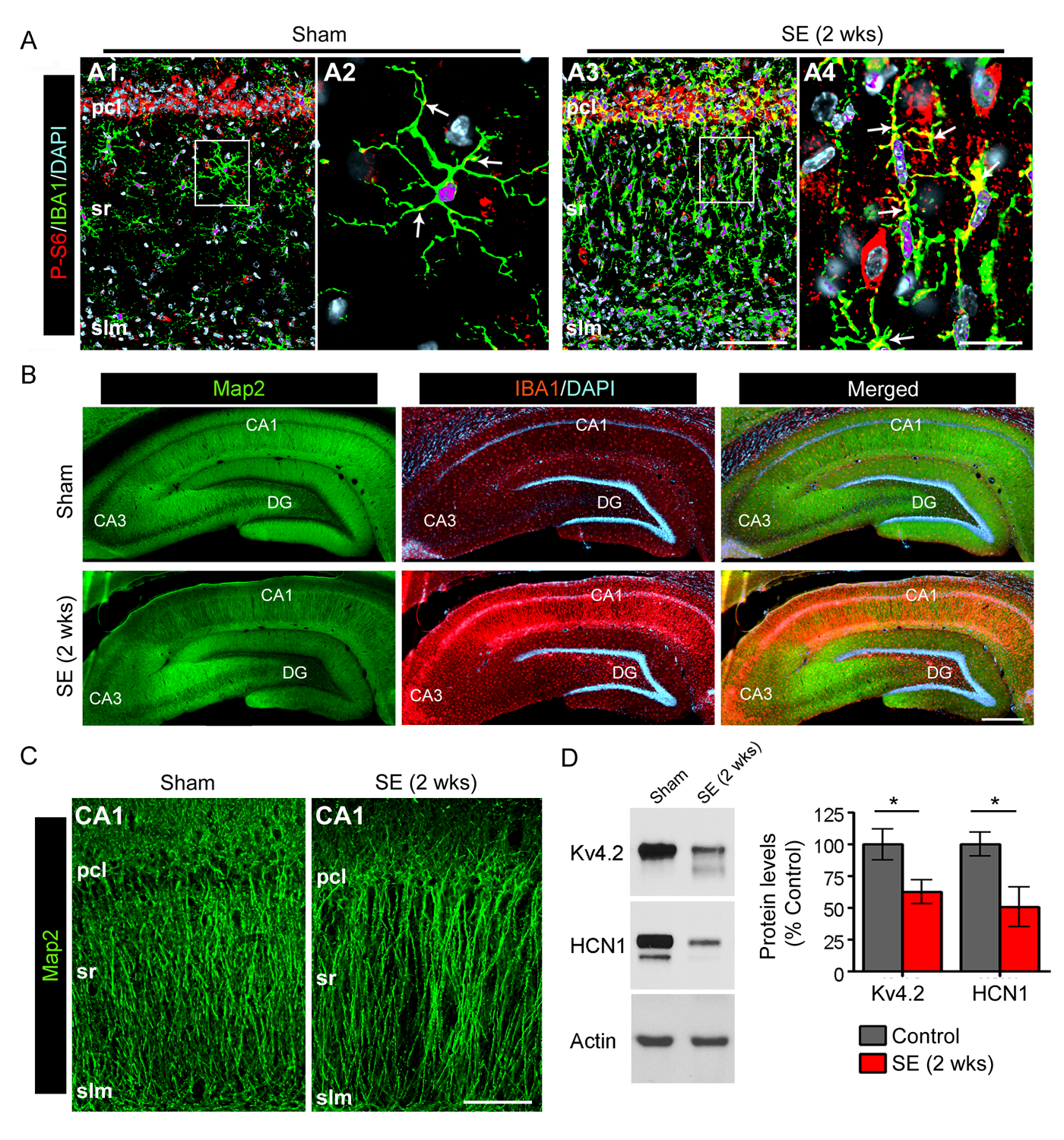

Supplement: Figure S4 — Reactive microglia expressed P-S6 and microgliosis correlated with Map2 loss in hippocampus from rats 2 weeks following SE. We used immunohistochemistry to evaluate P-S6, IBA1, and Map2 distribution in hippocampus from sham and SE rats two weeks after SE. (A) Representative low (A1, A3) and high (A2, A4) power images show IBA1 (green) and P-S6 (S240/244) (red) staining in the CA1 area of sham and SE (2 wks) rats. There was relatively greater P-S6 signal co-localized with IBA1 (yellow) (arrows) in stratum radiatum of the SE (2 wks) compared to the sham group, n = 3. (B) Representative low power images show representative hippocampal sections from sham and SE groups stained with antibodies against Map2 (green), IBA1 (red) and Dapi (blue). There is loss of Map2 staining and increased IBA1 signal in the CA1 region of the SE (2 wks) group compared to the sham group, n = 3. (C) High magnification images show loss of Map2 staining and Map2-labeled dendrites in the CA1 region of the SE (2 wks) compared with the sham group, n = 3. (D) Western blot analysis revealed significantly lower levels of Kv4.2 and HCN1 channel protein in the SE (2 wks) group compared with the sham group, n = 4−5. The deconvoluted maximum projection images shown in A1 and A3 are from 21 Z-stacks (0.5 µm steps), and those shown in A2 and A4 are from 13 Z-stacks (0.25 µm steps). Scale bars = A3,C: 100 µm; A4∶25 µm; B: 500 µm. Abbreviations: pcl, pyramidal cell layer; sr, stratum radiatum; slm, stratum lacunosum moleculare; DG, dentate gyrus; n = 3−4. (TIF) [file pone.0057808.s004.tif]

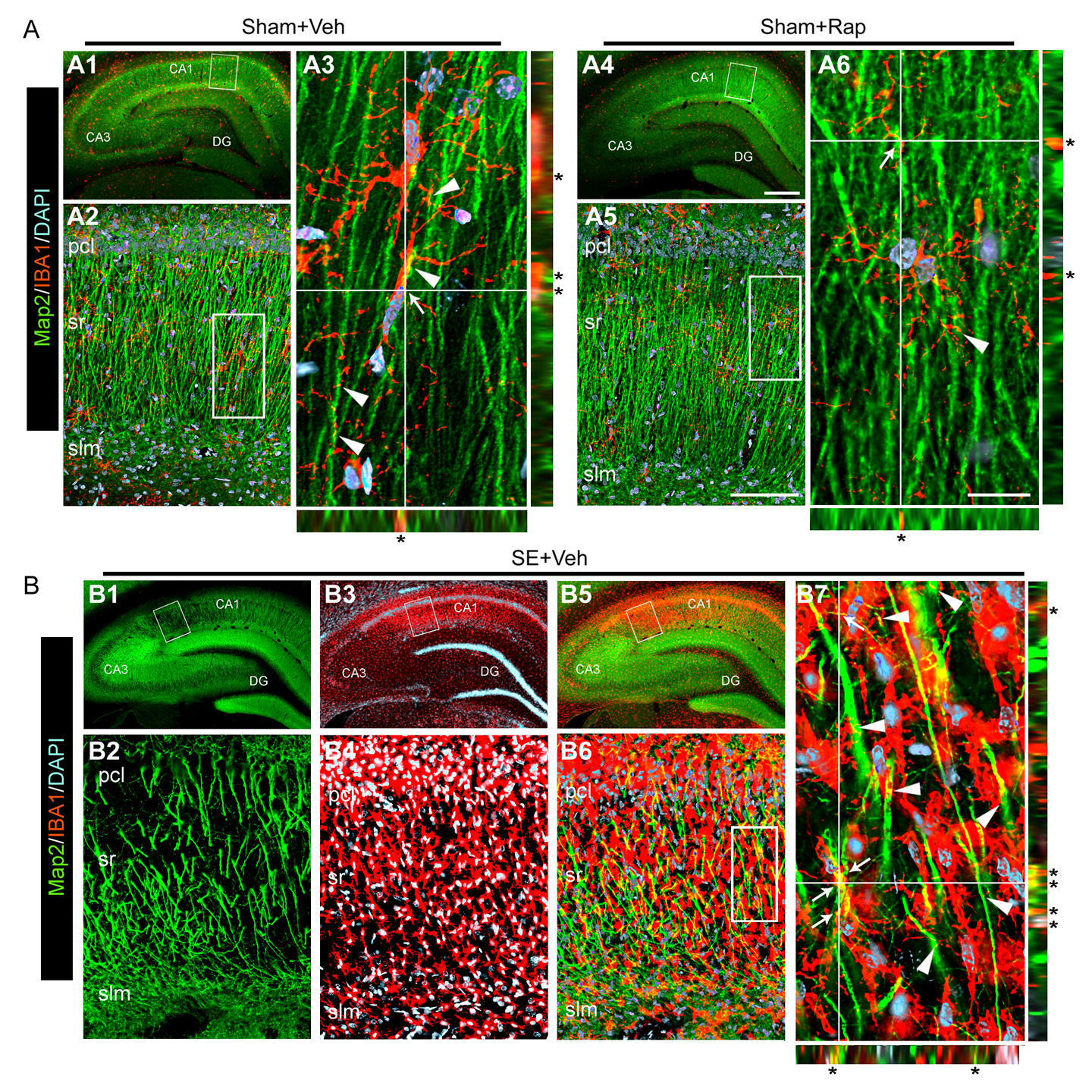

Supplement: Figure S5 — Rapamycin did not alter the organization and distribution of Map2 or IBA1 staining in the hippocampus of sham rats. We used immunohistochemistry to evaluate Map2 (green) and IBA1 (red) distribution in hippocampi from Sham+Veh (A1–3), Sham+Rap (A4–6) and SE+Veh rats (B). Dapi (blue) was used to stain cellular nuclei. (A) Representative low (A1, A4) and high (A2, A5) power images (boxed in top panels) are shown for the Sham+Veh and Sham+Rap groups, respectively. (A3, A6) Higher magnification images show microglial processes (red) intertwined with and touching (arrows and arrowheads) (yellow) the Map2-labeled dendrites (green) in these groups (area boxed in A2 and A5). This effect is evident in the XZ and YZ projections (arrows and asterisks). (B) Representative images from the SE+Veh group show a different CA1 region from the same hippocampus that is shown in figure 7D. (B7) The images show microglial processes intertwined with and touching the Map2-labeled dendrites in the SE+Veh group that is evident in the XZ and YZ projections (arrows and asterisks). The deconvoluted maximum projection images shown in A2, A5, B2, B5 and B6 are from 21 Z-stacks (0.5 µm steps), and those shown in A3, A6 and B7 are from 31 Z-stacks (0.1 µm steps). Scale bars: A4, B5∶500 µm; A5, B6∶100 µm; A6, B7∶25 µm. Abbreviations: pcl, pyramidal cell layer; sr, stratum radiatum; slm, stratum lacunosum moleculare; DG, dentate gyrus; n = 4−6. (TIF) [file pone.0057808.s005.tif]
