# Supplementary material for: Rapamycin Reverses Status Epilepticus-Induced Memory Deficits and Dendritic Damage
Source: PLoS One. 2013 Mar 11;8(3):e57808. doi: 10.1371/journal.pone.0057808 (PMC3594232; doi:10.1371/journal.pone.0057808)
Supplement: Methods S1 — Supporting Methods. (DOCX) [file pone.0057808.s006.docx]

**Supporting Methods**

*Open field:* The animals were tested in an open-field arena to measure locomotor activity. The mice were weighed and allowed to acclimatize to the testing room for 30 min. Then the rats were placed into the center of a clear Plexiglas (40×40×30 cm) open-field arena and allowed to explore for 30 min. The lighting inside the test chamber was approximately 100 lux. The movement of the rats was monitored by a video camera connected to a digital tracking device (Noldus Information Technology, Leesburg, VA, USA). We measured total distance moved and the velocity (speed) of the animal. We also measured the time in the center in the open field and frequency of transitions between the center and outer portions of the open-field to measure changes in anxiety-like behavior. Between each trial, the area was thoroughly cleaned with a 30% isopropyl alcohol then dried with paper towels.

*Social behavior:* The social behavior of the rats was measured in the open field arena (40×40×30 cm). Social partners were age, weight, and sex-matched to the experimental animals. Rats were habituated to the arena for 5 minutes 24 h before testing. On the day of testing, the experimental animals were first introduced into the arena for one minute then the social partner was introduced. The rats’ behavior in the test arena was recorded for 10 min. Then the frequency and time of active social behavior was scored. Active social behaviors consisted of anogenital investigation, non-anogenital investigation, social exploration, chasing, and grooming of the social partner. Between each trial, the area was thoroughly cleaned with a 30% isopropyl alcohol then dried with paper towels.
